# Dynamic assessment of renal resistive index using point-of-care ultrasonography for early detection of sepsis-induced acute kidney injury: A multicenter clinical trial protocol

**DOI:** 10.1371/journal.pone.0359982

**Published:** 2026-10-29

**Authors:** Zongfang Ren, Lingling Wang, Jinxi Yue, Nimei Xu, Ming Wu, Zhifeng Liu

**Affiliations:** 1 The Department of Critical Care Medicine of the Second Affiliated Hospital of Kunming Medical University, Kumming, China; 2 The First School of Clinical Medicine, Southern Medical University, Guangzhou, China; 3 Department of Medical Intensive Care Unit, General Hospital of Southern Theater Command, Guangzhou, China; 4 Department of Critical Care Medicine, Jiangmen Central Hospital of Guangdong Province, Jiangmen, China; 5 Department of Infection and Critical Care Medicine, Shenzhen Second People’s Hospital & First Affiliated Hospital of Shenzhen University, Shenzhen, China; Christian Medical College Vellore, INDIA

## Abstract

**Background:**

Acute kidney injury (AKI) is a common and serious complication of sepsis, significantly increasing both mortality risk and healthcare costs. Early prediction and diagnosis of sepsis-associated AKI (SA-AKI) remain challenging due to the delayed detection of existing renal injury markers. Although research suggests that the Renal Resistive Index (RRI) shows promise for earlier prediction of AKI, large-scale, multicenter clinical studies to support this are currently lacking.

**Methods:**

This multicenter, open-label, prospective observational cohort study enrolled 238 patients who were diagnosed with sepsis according to the Sepsis 3.0 criteria. The Renal Resistive Index (RRI) was measured using bedside ultrasound within 2 hours of diagnosis and subsequently every 8 hours during their individualized standard treatment. Concurrently, blood samples were collected to quantify creatinine and cystatin C, and urine samples were obtained to detect kidney injury markers, including TIMP-2/IGFBP-7. The primary endpoint is to determine the threshold and accuracy of the Renal Resistive Index in predicting sepsis-associated acute kidney injury (SA-AKI). Secondary endpoints include examining the dynamic trend of the RRI, assessing the RRI's predictive value for 28-day mortality in sepsis, and evaluating the correlation between the RRI and creatinine, cystatin C, and urinary TIMP-2/IGFBP-7 levels.

**Discussion:**

This study assesses the accuracy and feasibility of using bedside ultrasonography to monitor the Renal Resistive Index (RRI) for the early detection of sepsis-associated acute kidney injury (SA-AKI). Early identification of SA-AKI is crucial for improving clinical outcomes in sepsis, making this approach highly relevant for timely intervention.

**Ethics and Dissemination:**

This study received ethical approval from the Ethics Committee of the initiator's institution. Study results will be initially reported to the collaborating investigators. Following review and consensus by the Trial Steering Committee, the research report will be drafted by the initiating investigator and disseminated through peer-reviewed publications and presentations at scientific conferences.

**Trial registration:**

Chinese Clinical Trial Registry (ChiCTR2400082420), Registered on March 28, 2024.

## Introduction

Sepsis, defined as a life-threatening organ dysfunction caused by a dysregulated host response to infection, is frequently complicated by acute kidney injury (AKI) [1,2].This is the most prevalent complication of sepsis, with incidence rates ranging from 45% to 70%. Sepsis-associated AKI (SA-AKI) is a critical determinant of high mortality rates and accounts for substantial medical expenses [3]^.^ Research has consistently demonstrated that the development of SA-AKI is associated with a worse prognosis, including longer lengths of stay in intensive care units (ICUs) and hospitals. Furthermore, it is a major contributor to long-term functional impairment and reduced quality of life among sepsis survivors [4]. Despite its significant impact, the absence of effective methods for the early prediction and identification of AKI poses a major challenge to clinicians in the current clinical setting and is a principal reason why AKI often fails to receive timely intervention.

Current understanding suggests that the main pathogenic mechanisms underlying SA-AKI involve endothelial cell injury within renal tissue, coupled with the activation of leukocytes and platelets to form microthrombi, ultimately leading to renal microcirculation disorders [4,5]. An increase in renal vascular resistance is posited to be a crucial hemodynamic factor implicated in SA-AKI [6,7]. While serum creatinine and urine output are clinical parameters widely used to evaluate renal function, they are indirect indicators reflecting physiological processes and typically manifest abnormalities only in the late stages of AKI, exhibiting relatively low sensitivity. Therefore, exploring alternative detection methods capable of earlier prediction and assessment of SA-AKI is of significant clinical importance.

The Renal Resistive Index (RRI), an indicator derived from Doppler ultrasound technology by measuring the blood flow spectrum of intrarenal arteries (such as interlobar or arcuate arteries), is a non-invasive parameter used to assess renal vascular resistance and parenchymal perfusion status [8–10]. As a non-invasive and repeatable bedside monitoring parameter of renal hemodynamics, RRI has been applied in the early prediction and evaluation of AKI in other contexts, such as after severe trauma or related to COVID-19 infection [11]. However, despite its potential, there remains a notable lack of large-scale, multicenter cohort studies systematically evaluating the clinical application value of RRI specifically in sepsis-associated acute kidney injury (SA-AKI).

### Definitions

SA-AKI is defined as the occurrence of AKI within 7 days of sepsis onset (diagnosed according to Kidney Disease Improving Global Outcome Criteria and Sepsis 3 criteria, respectively). Early SA-AKI means AKI occurring within 48 hours of sepsis diagnosis [12].

Baseline serum creatinine was defined as follows: For patients with recent laboratory data (within 3 months prior to admission), the most recent pre-admission serum creatinine value was used. For patients without recent measurements, baseline creatinine was estimated using the Modification of Diet in Renal Disease (MDRD) equation, assuming a normal baseline eGFR of 75 mL/min/1.73 m^2^,This approach follows the KDIGO 2012 recommendations [13].

Baseline RRI is the RRI value measured at enrollment, serving as the reference point for analyses during the study period; Maximum RRI is the highest RRI value observed among all measurements during the study period, used to assess the most severe renal vascular resistance state of the patient; Mean RRI is the arithmetic mean of all RRI measurements during the study period, reflecting the overall trend of the patient’s renal vascular resistance; Change in RRI is the difference between RRI values during the study period and the baseline RRI, used to evaluate dynamic changes in renal vascular resistance.

### Study objectives

#### Primary objective.

The primary objective of this study is to evaluate the predictive value of dynamic monitoring of the Renal Resistive Index (RRI) for the early development of sepsis-associated acute kidney injury (SA-AKI).

#### Secondary objectives.

1. To assess the feasibility and reliability of implementing serial RRI measurements in critically ill septic patients at the bedside.
2. To determine optimal RRI threshold values for SA-AKI risk stratification, utilizing receiver operating characteristic (ROC) curve analysis.
3. To investigate the correlation between RRI values and established and novel biomarkers of kidney injury (serum creatinine, cystatin C, urinary TIMP-2/IGFBP-7).
4. To evaluate the prognostic significance of RRI for predicting 28-day mortality and the need for renal replacement therapy in septic patients

#### Methods and analysis.

This protocol was developed in accordance with the STROBE (Strengthening the Reporting of Observational Studies in Epidemiology) guidelines for reporting observational studies [14]. Following the completion of the preliminary standardized training, the recruitment period for this study is set for December 2025 to December 2027. The study flowchart is shown in Fig 1.

**Fig 1:**
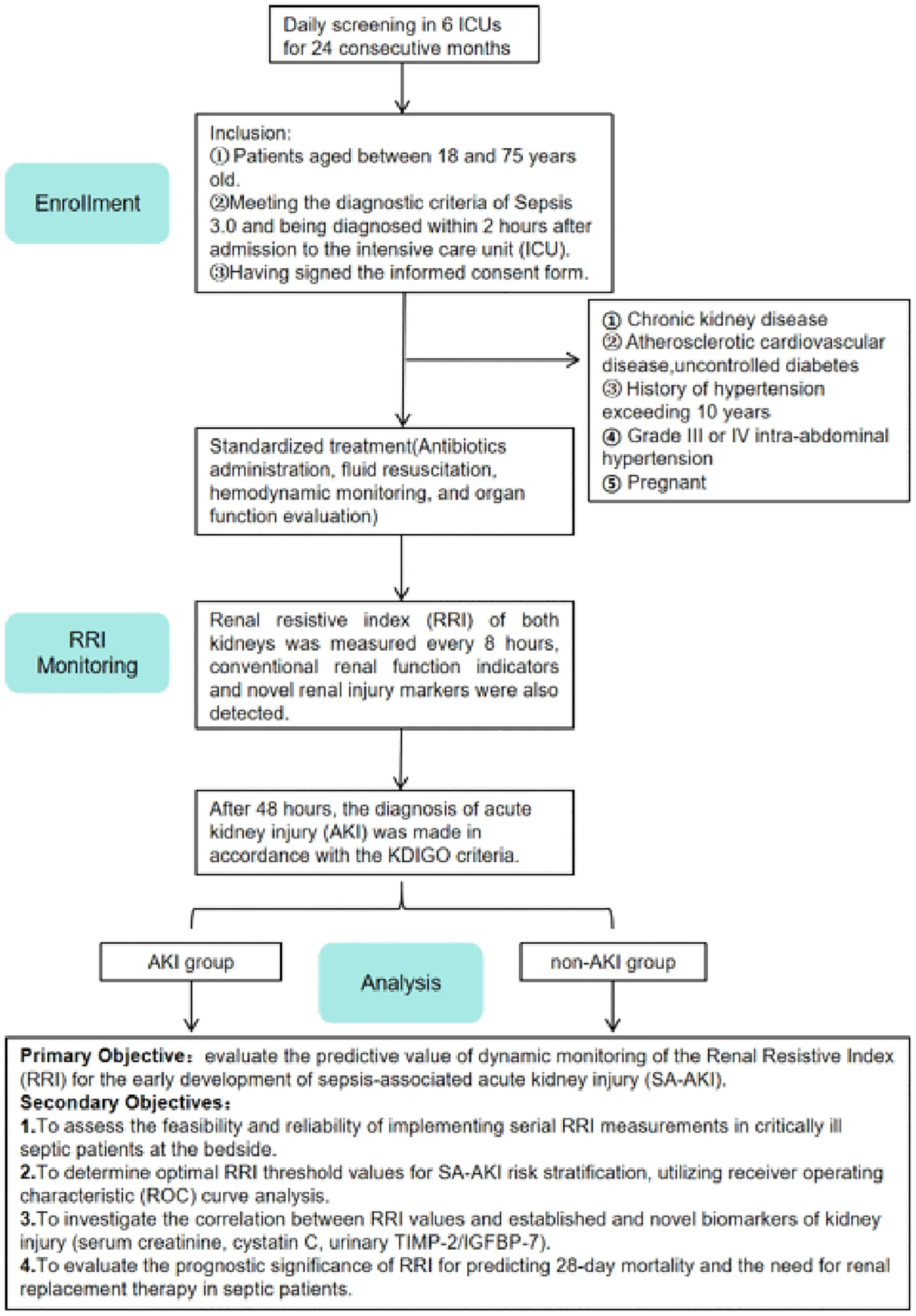
The flow chart of the study.

#### Trial design.

This multicenter, prospective observational cohort study enrolled 238 patients who were diagnosed with sepsis according to the Sepsis 3.0 criteria. The study was initiated by the General Hospital of the Southern Theater Command of the People's Liberation Army (PLA). A Data Quality Monitoring Committee (DQMC) was established to provide periodic review and evaluation of trial data from each participating center. The trial design and reporting adhere to the STROBE (Strengthening the Reporting of Observational Studies in Epidemiology) guidelines [14].

#### Trial setting.

It will be conducted in the Intensive Care Units (ICUs) of six large tertiary teaching hospitals across mainland China.The participating ICUs were staffed by professional critical care physicians and nurses, ensuring the provision of standardized treatment for sepsis. Furthermore, these units were equipped with intelligent central monitoring and data acquisition systems, as well as central laboratories capable of separating, preserving, and transporting plasma to a central analysis facility.

#### Standardization and reliability of RRI measurements.

Ultrasound examinations in this study were performed by board-certified intensive care unit (ICU) physicians who had undergone rigorous competency-based training. All images were independently reviewed by two readers to ensure consistent interpretation. All participating centers adhered to a standardized protocol, including probe position, angle, and respiratory phase, to maintain comparability across repeated measurements in this multicenter study. Each image was independently evaluated by two readers, with inter- and intra-observer reliability assessed using the intraclass correlation coefficient (ICC) for continuous variables and Cohen’s κ coefficient for categorical variables, ensuring the consistency and reliability of measurements.Typical ultrasound images are shown in Fig 2.

**Fig 2:**
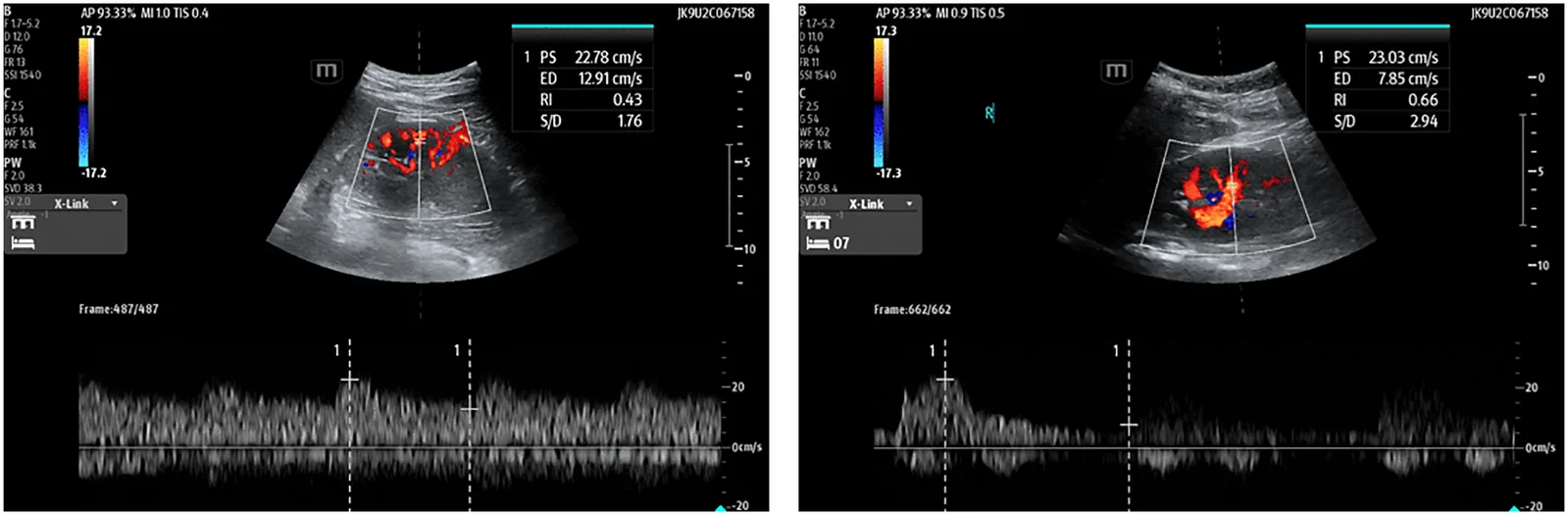
Evaluation of the renal resistive index using Doppler ultrasound. The transducer is placed in an interlobar artery, and the spectral Doppler examines the peak systolic and end-diastolic velocities.

#### Inclusion criteria.

Patients will be considered eligible for inclusion in this trial if they meet all of the following criteria.

1. Age between 18 and 75 years, inclusive.
2. Meeting the Sepsis 3.0 diagnostic criteria and being diagnosed within 2 hours of admission to the intensive care unit (ICU).
3. Provision of written informed consent by the patient or their legally authorized representative

#### Exclusion criteria.

Patients meeting any of the following criteria will be excluded from participation.

1. Advanced chronic kidney disease (CKD stages 3–5 based on Kidney Disease: Improving Global Outcomes [KDIGO] guidelines).
2. Known atherosclerotic cardiovascular disease or uncontrolled diabetes mellitus requiring ongoing insulin therapy.
3. History of poorly controlled hypertension for more than 10 years despite triple antihypertensive therapy, or chronic heart failure classified as New York Heart Association (NYHA) Class III or IV.
4. Intra-abdominal hypertension Grade III or IV (defined as intra-abdominal pressure≥21 mmHg according to the World Society of the Abdominal Compartment Syndrome criteria).
5. Patients with a clinically estimated mortality risk exceeding 90% within the next 48 hours in the ICU, as determined by the Acute Physiology and Chronic Health Evaluation (APACHE) II score.
6. Confirmed pregnancy via serum β-human chorionic gonadotropin (β-HCG) testing and ultrasound examination.
7. Patients for whom inclusion is deemed inappropriate by the principal investigator due to unresolved ethical concerns, designation for palliative care, or issues related to legal guardianship.

#### Criteria for participant withdrawal.

Participants may be withdrawn from the trial for any of the following reasons. The date, reason, and procedure for withdrawal will be meticulously documented in the participant's case report form.

1. Significant non-compliance with the study protocol.
2. Voluntary request for withdrawal by the participant or their legally authorized representative.
3. Development of a serious adverse event requiring discontinuation of study procedures.
4. Other circumstances which, in the opinion of the investigator, make continuation in the trial not in the best interest of the participant or impractical.

### Informed consent

Prior to the initiation of this trial, a comprehensive assessment of participant safety was conducted during protocol design. Bedside ultrasound, as a non-invasive procedure, is not anticipated to cause irreversible or severe harm to participants. Investigators are obligated to clearly and thoroughly explain the trial objectives, potential risks, anticipated discomforts, and corresponding management strategies to participants or their legally authorized representatives. Confirmation of full comprehension of this information must be obtained. Following the provision of explicit consent, participants or their legally authorized representatives are required to sign and date the written informed consent form, thereby formally documenting their willingness to participate in this trial.

#### Sample size estimation.

Based on results from our pilot study, we assumed a sensitivity of 85% and a specificity of 80% for RRI in predicting sepsis-associated acute kidney injury (SA-AKI), with an expected SA-AKI incidence of 55%. Sample size calculation was performed using the area under the receiver operating characteristic curve (AUC) method to comprehensively reflect the predictive performance of RRI across all possible thresholds. Under a two-sided α of 0.05 and a precision of ±8%, the estimated sample size was 214 patients. Accounting for an anticipated 10% rate of missing data, the final sample size was increased to 238 patients. For this multicenter study involving six centers, this corresponds to approximately 40 patients per center.

#### Patient and public involvement statement.

The study not appropriate to involve patients or the public in the design, or conduct, or reporting, or dissemination plans of our research.

### Study procedure

#### Standardized treatment.

All enrolled patients received comprehensive interventions strictly adhering to the 2026 Surviving Sepsis Campaign (SSC) guidelines for standardized management. Patients diagnosed with septic shock, according to the Sepsis 3.0 criteria, received immediate administration of early goal-directed therapy (EGDT). Continuous hemodynamic monitoring was implemented, including assessment of central venous pressure (CVP), mean arterial pressure (MAP), urine output (UO), central venous oxygen saturation (ScvO2), and lactate levels. The monitoring protocol was established by a board-certified critical care physician with over five years of specialized intensive care experience.

#### Renal resistive index and renal function monitoring.

Following confirmation of sepsis diagnosis, all patients underwent immediate bedside renal Doppler ultrasonography monitoring. Using a 5–2 MHz convex probe, the Renal Resistive Index (RRI) was measured bilaterally at the interlobar or arcuate arteries.To ensure consistency among all centers and operators, the measurement was conducted at the arcuate artery located at the junction of the cortex and medulla. Measurements were performed at 8-hour intervals throughout a 7-day monitoring period, with mean values recorded from three cardiac cycles per kidney. Concurrently, daily venous blood samples were collected for quantitative analysis of serum creatinine (SCr), cystatin C (CysC). First-morning midstream urine specimens were routinely obtained for determining the ratio of tissue inhibitor of metalloproteinase-2 (TIMP-2) to insulin-like growth factor binding protein-7 (IGFBP-7).The diagnostic criteria for AKI are presented in Table 1.

**Table 1:** Acute kidney injury (AKI) diagnostic criteria.

AKI Diagnostic Criteria	
Stage	Serum Creatinine and Urine Output
Ⅰ	Increase in SCr of 1.5–1.9 times baseline or ≥0.3 mg/dL. Urine output <0.5 mL/kg/hr for 6–12 hours.
Ⅱ	Increase in SCr of 2.0–2.9 times baseline. Urine output <0.5 mL/kg/hr for ≥12 hours.
Ⅲ	Increase in SCr of ≥3.0 times baseline or SCr ≥ 4.0 mg/dL (≥354 µmol/L), or initiation of renal replacement therapy (RRT). Urine output <0.3 mL/kg/hr for ≥24 hours, or anuria for ≥12 hours

#### Routine monitoring protocol.

Routine monitoring was conducted throughout the study period. This included daily assessment of hematologic parameters: white blood cell count (WBC), neutrophil percentage (NEUT%), and platelet count (PLT). Coagulation parameters, including prothrombin time (PT), activated partial thromboplastin time (APTT), international normalized ratio (INR), fibrinogen (FIB), and fibrinogen degradation products (FDP), were evaluated at 24-hour intervals. Biochemical markers, such as hepatic function (alanine aminotransferase [ALT], aspartate aminotransferase [AST]), electrolytes (potassium [K], sodium [Na], chloride [Cl]), and blood glucose (Glu), were also monitored every 24 hours.

#### Hemodynamic monitoring.

All enrolled patients were monitored for mean arterial pressure (MAP) and central venous pressure (CVP), and the type, dosage, and duration of vasoactive medications used were recorded.

#### Mechanical ventilation monitoring.

For patients receiving mechanical ventilation, the duration of ventilator use, positive end-expiratory pressure (PEEP), and oxygenation index (PaO_2_/FiO_2_) were recorded.

### Outcomes

#### Primary outcome.

The primary outcome is the identification of the optimal threshold of the renal resistive index (RRI) for predicting acute kidney injury (AKI) occurring within 48 hours of sepsis diagnosis, assessed by its corresponding sensitivity, specificity, and the area under the receiver operating characteristic curve (AUC).

#### Secondary outcomes.

1. Assessment of the longitudinal trajectory of RRI values throughout the disease course in septic patients.
2. Evaluation of the correlation coefficients between RRI and various biomarkers. ①Serum biomarkers:creatinine (Cr) and blood urea nitrogen (BUN). ②Tubular injury markers:cystatinC (CysC) ③Urinary cell cycle arrest biomarkers:the ratio of tissue inhibitor of metalloproteinase-2 (TIMP-2) to insulin-like growth factor binding protein-7 (IGFBP-7).
3. Determination of the prognostic value of RRI for 28-day clinical outcomes, including mechanical ventilation duration, requirement for renal replacement therapy, and mortality.

### Data collection and management

#### Data sources and recording.

Data for this study were acquired through the Clinical Electronic Information System (CEIS) and recorded on standardized Case Report Forms (CRFs). To ensure data quality, two independent data reviewers were appointed at each participating center. These reviewers performed a dual-review of the CRF data to guarantee accuracy, integrity, and timeliness. All completed CRFs will be stored electronically to facilitate subsequent statistical analysis and data management.The study period and clinical data collection are shown in Fig 3.

**Fig 3:**
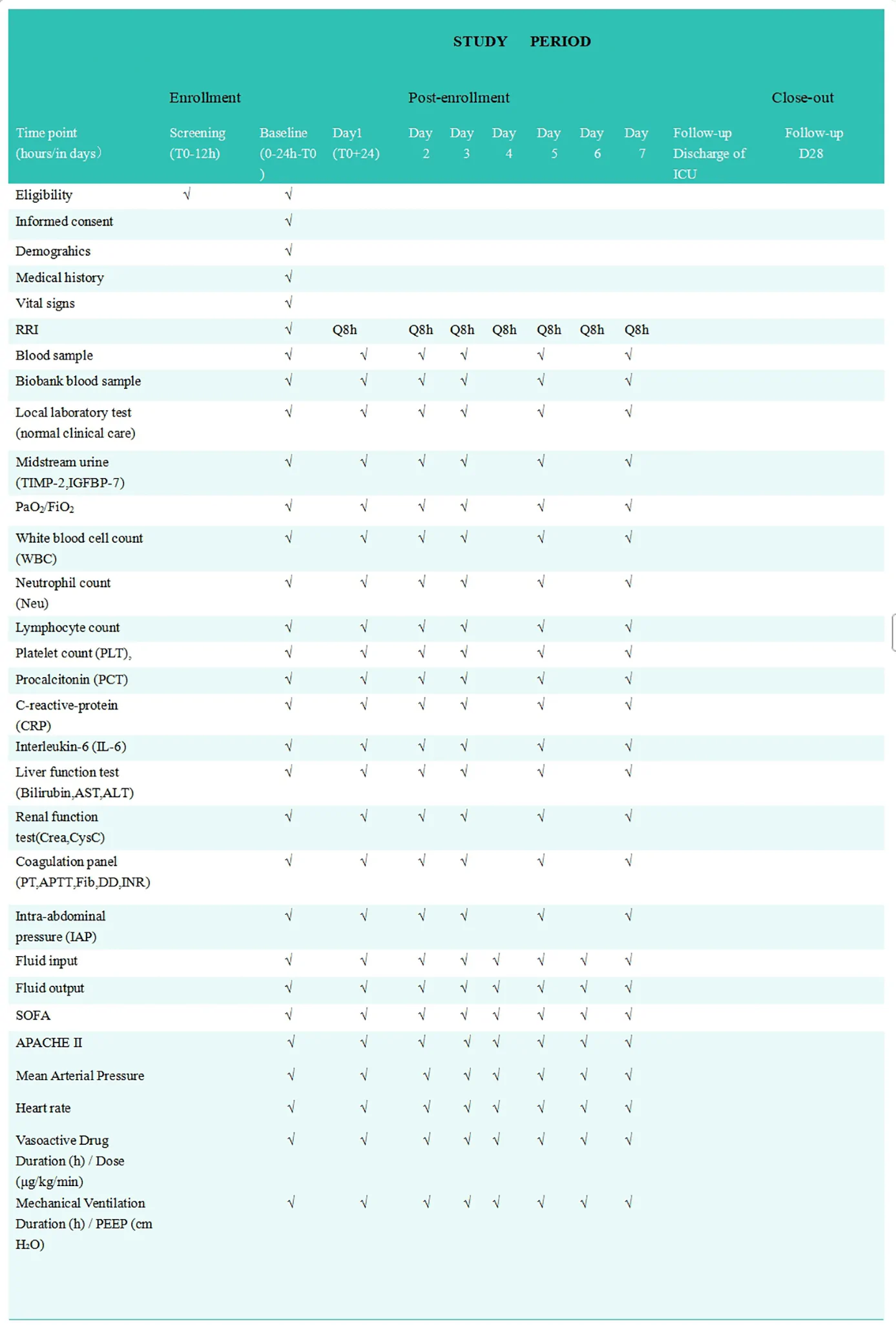
Study period.

#### Baseline data collection.

Upon patient enrollment, comprehensive baseline data will be collected. These data include, but are not limited to, demographic characteristics (age, sex), history of comorbid conditions, primary diagnosis, and relevant severity scoring metrics such as the Acute Physiology and Chronic Health Evaluation II (APACHE II) and the Sequential Organ Failure Assessment (SOFA). Additionally, details regarding the site of infection and identified pathogen information will be recorded.

#### Dynamic monitoring data collection.

The following dynamic monitoring data will be collected and documented on days 1, 2, 3, 5, and 7 following patient enrollment:

**Vital signs:** Heart rate, blood pressure, respiratory rate, and body temperature.

**Laboratory testing:** White blood cell count (WBC), neutrophil count, lymphocyte count, platelet count (PLT), procalcitonin (PCT), C-reactive protein (CRP), and interleukin-6 (IL-6).

**Biochemical parameters:** Hepatic function (alanine aminotransferase [ALT], aspartate aminotransferase [AST]), renal function (serum creatinine [SCr], blood urea nitrogen [BUN]), oxygenation index (PaO_2_/FiO_2_), and lactate level.

**Coagulation panel:** Prothrombin time (PT), activated partial thromboplastin time (APTT), international normalized ratio (INR), fibrinogen (FIB), and D-dimer (DD).

**Special Monitoring and Fluid Management:** Intra-abdominal pressure (IAP) measurements (performed every eight hours and documented on these specified days).

24-hour fluid input and output (recorded daily and documented on these specified days). Fluid input encompasses crystalloids, colloids, albumin, and blood products. Fluid output includes urine volume and other drainage volumes. The 24-hour fluid balance will subsequently be calculated. Mean arterial pressure (MAP), vasopressor use including dosage and duration, duration of mechanical ventilation, and positive end-expiratory pressure (PEEP) were also dynamically monitored and recorded.

### Confidentiality

The findings of this research project may be published in medical journals. However, the confidentiality of patient information will be strictly maintained in full compliance with all applicable legal and regulatory requirements. Patient personal information will not be disclosed or used for any purpose other than that explicitly outlined in this protocol, except where mandated by law. Authorized representatives of governmental regulatory authorities, institutional ethics committees, and the sponsor may be permitted to review patient records and source data for monitoring and auditing purposes, to the extent necessary to fulfill their regulatory responsibilities, while maintaining confidentiality. It is emphasized that access to confidential information will be strictly limited to authorized personnel for legitimate purposes.

### Statistical analysis

Continuous variables will be presented as the mean and standard deviation (SD) for normally distributed data, or as the median and interquartile range (IQR) for non-normally distributed data. Categorical variables will be summarized as frequencies and percentages.To analyze the relationship between the Renal Resistive Index (RRI) and 28-day mortality, a Cox proportional hazards model will be employed, with adjustments made for potential confounding variables such as center and age. Survival probabilities will be estimated using Kaplan-Meier curves, and differences in survival between distinct RRI groups will be compared using the log-rank test.The dynamic changes in RRI over time will be analyzed using a mixed-effects model for repeated measures.This model will account for within-subject variability and the influence of fixed effects, including time points, center, and age, to assess the trajectory of RRI values. The predictive performance of the Renal Resistive Index (RRI) for acute kidney injury (AKI) occurring within 48 hours of sepsis diagnosis will be evaluated using receiver operating characteristic (ROC) curve analysis. The optimal RRI threshold will be determined using the Youden Index. The 95% confidence interval for the sensitivity and specificity associated with the optimal threshold will be calculated using the Clopper-Pearson exact method.All statistical analyses will be performed using SPSS25 (IBM Corp., Armonk, NY, USA), A two-tailed p-value of < 0.05 will be considered statistically significant.

In the multivariable models, we will include the following hemodynamic variables: mean arterial pressure (MAP), vasopressor use and dosage, mechanical ventilation status, PEEP, intra-abdominal pressure, vascular stiffness, age, and fluid balance. These covariates will be adjusted for to account for potential confounding and to ensure that changes in RRI predominantly reflect renal microcirculatory alterations rather than systemic hemodynamic fluctuations.

In the primary analysis of this study, mean renal resistive index (Mean RRI) will be used as the main predictor. Maximum RRI during follow-up, baseline RRI, and changes in RRI over time will be included as secondary analyses to evaluate dynamic trends. The estimated AKI incidence is approximately 55%, corresponding to around 130 events. To minimize overfitting, the final models will include only 8–10 key covariates (with a maximum of 13), all selected based on clinical relevance and prior literature, ensuring sufficient events per variable (EPV ≥ 10). Model robustness will be assessed using cross-validation or bootstrap methods to further reduce the risk of overfitting.

### Handling of missing data

During the trial, every effort will be made to minimize missing data, particularly for primary and secondary outcome measures. In the event of missing values, multiple regression imputation will be used to handle the missing data. Patients with five or more missing measurements will be excluded from the analysis and not included in the statistical models.

## Discussion

The incidence of sepsis-associated acute kidney injury (SA-AKI) is high. Currently, the commonly used clinical indicators for diagnosis are often delayed. Researchers have been constantly searching for biomarkers that can predict AKI at an early stage. The Renal Resistive Index (RRI) provides a comprehensive reflection of alterations in renal microcirculation and can predict the onset of AKI at an early stage. Early detection of elevated RRI in septic patients may enable clinicians to anticipate AKI before changes in traditional biochemical markers occur. This allows for timely interventions, including hemodynamic optimization, adjustments in fluid management, and avoidance of nephrotoxic agents. By identifying patients at high risk sooner, RRI measurement can inform clinical decision-making and potentially improve patient outcomes. Furthermore, RRI offers a non-invasive, bedside tool that can complement conventional monitoring. Overall, incorporating RRI into early assessment strategies may enhance the precision of AKI risk stratification in sepsis.

However, to date, no large-scale multicenter studies have been carried out among sepsis patients. The main reason might be that critically ill patients in the intensive care unit (ICU) face difficulties in being transported to the imaging diagnosis center for RRI measurement due to their critical conditions. With the widespread application of portable bedside ultrasound in the ICU, the dynamic monitoring of RRI has emerged as a highly convenient technique. This development is conducive to the implementation of this trial.

The mortality rate among patients with sepsis complicated by acute kidney injury (AKI) remains alarmingly high. This elevated mortality can be attributed, in part, to our limited understanding of the pathophysiological mechanisms underlying sepsis-associated AKI [15–17]. Recent experimental investigations have revealed that the pathogenesis of sepsis-induced AKI is considerably more intricate than mere renal hypoperfusion resulting from reduced cardiac output and hypotension. Renal microvascular alterations induced by sepsis, such as vasoconstriction, capillary leak syndrome accompanied by tissue edema, leukocyte and platelet adhesion associated with endothelial dysfunction and/or microthrombus formation, and/or an increase in intra-abdominal pressure, may contribute to an elevation in renal vascular resistance (RVR) [18–20]. Additional research is essential to elucidate the temporal sequence of renal microvascular changes during sepsis, as well as the onset and progression of acute kidney injury (AKI). Doppler ultrasonography, in conjunction with the calculation of the resistance index, can provide insights into the extent of vascular resistance alterations. This approach is instrumental in predicting the development of persistent AKI and identifying the optimal systemic hemodynamics necessary to sustain renal perfusion.

A prospective observational study conducted by Mahmoud Marzouk et al. involving 40 patients with septic shock demonstrated that the Renal Resistive Index (RRI) could serve as an early predictor of acute kidney injury (AKI). Continuous and dynamic monitoring of RRI may play a critical role in guiding the management of septic shock [10]. Additionally, a clinical study performed by Roberta Barone et al. with 148 participants revealed that RRI is independently associated with renal dysfunction in patients undergoing coronary angiography [21]. RRI provides a more precise reflection of renal pathophysiological alterations, hemodynamic status, and functional reserve [22]. In this trial, we aim to broaden the scope of our investigation by conducting a multicenter, prospective cohort study. Through dynamic RRI monitoring and concurrent detection of both conventional and novel kidney injury biomarkers, we seek to identify optimal indicators and assessment techniques for the early prediction of sepsis-related AKI, thereby offering valuable insights and practical guidance for clinicians.

It is noteworthy that in the ICUs of large tertiary hospitals in China, senior physicians typically conduct rounds every 8 hours. Therefore, in this study, the RRI measurement interval was set at 8 hours to align with clinical workflows while minimizing interference with routine patient care.

### Trial status

Recruitment for this trial commenced on June 12, 2024, and is anticipated to conclude by December 31, 2026.
