# Supplementary material for: Dynamic assessment of renal resistive index using point-of-care ultrasonography for early detection of sepsis-induced acute kidney injury: A multicenter clinical trial protocol
Source: PLoS One. 2026 Oct 29;21(10):e0359982. doi: 10.1371/journal.pone.0359982 (PMC13718935; doi:10.1371/journal.pone.0359982)
Supplement: Supplementary file 1 [file pone.0359982.s001.pdf]

PLOS ONE

Dynamic Assessment of Renal Resistive Index Using Point-of-Care Ultrasonography for Early Detection of Sepsis-Induced Acute Kidney Injury: A Multicenter Clinical Trial Protocol

--Manuscript Draft--

**Manuscript Number:**	PONE-D-25-46681
**Article Type:**	Study Protocol
**Full Title:**	Dynamic Assessment of Renal Resistive Index Using Point-of-Care Ultrasonography for Early Detection of Sepsis-Induced Acute Kidney Injury: A Multicenter Clinical Trial Protocol
**Short Title:**	Runing title: Early Predictive Value of Renal Resistive Index for Sepsis-associated Acute Kidney Injury
**Corresponding Author:**	zongfang Ren<br>Kunming Medical University Second Affliated Hospital<br>KunMing, YunNan CHINA
**Keywords:**	Acute kidney injury; sepsis; Renal Blood Flow Index; clinical trial
**Abstract:**	Background<br><br>Acute kidney injury (AKI) is a major complication of sepsis, markedly increasing mortality risk and healthcare costs. Early prediction and diagnosis of sepsis-associated AKI (SA-AKI) are difficult due to the delayed nature of existing renal injury markers. While research indicates the renal resistive index (RRI) holds promise for earlier AKI prediction, this is currently unsupported by large-scale, multicenter clinical studies.<br><br>Methods<br><br>This study is a multicenter, open-label, non-blinded clinical trial enrolling 238 patients diagnosed with sepsis according to the Sepsis 3.0 criteria. The renal resistive index (RRI) was measured using bedside ultrasound within 2 hours after diagnosis and subsequently every 8 hours throughout their individualized standard treatment. Simultaneously, blood samples were collected for the quantification of creatinine and cystatin C, and urine samples were obtained for the detection of kidney injury markers, including TIMP-2/IGFBP-7. The primary endpoint is to establish the threshold and accuracy of the renal resistive index in predicting sepsis-associated acute kidney injury (AKI). Secondary endpoints include exploring the dynamic trend of the renal resistive index; assessing the predictive value of the renal resistive index for 28-day mortality in sepsis; and evaluating the correlation between the renal resistive index and creatinine, cystatin C, and urinary TIMP-2/IGFBP-7.<br><br>Discussion<br><br>This study evaluates the accuracy and feasibility of bedside ultrasonography-based monitoring of the Renal Resistive Index (RRI) for the early detection of sepsis-associated acute kidney injury (AKI), given that timely identification is critical for improving clinical outcomes in sepsis.<br><br>Ethics and Dissemination<br><br>This study received ethical approval from the Ethics Committee of the initiator's institution. Study results will be initially reported to the collaborating investigators. Following review and consensus by the Trial Steering Committee, the research report will be drafted by the initiating investigator and disseminated through peer-reviewed publications and presentations at scientific conferences.
**Order of Authors:**	zongfang Ren
	Lingling Wang
	Jinxi Yue

	Nimei Xu
	M Wu
	Zhifeng Liu
**Additional Information:**	
**Question**	**Response**
**Financial Disclosure**<br><br>Enter a financial disclosure statement that describes the sources of funding for the work included in this submission. Review the submission guidelines for detailed requirements. View published research articles from *PLOS ONE* for specific examples.<br><br>This statement is required for submission and **will appear in the published article** if the submission is accepted. Please make sure it is accurate.<br><br>**Funded studies**<br>Enter a statement with the following details:<br>• Initials of the authors who received each award<br>• Grant numbers awarded to each author<br>• The full name of each funder<br>• URL of each funder website<br>• Did the sponsors or funders play any role in the study design, data collection and analysis, decision to publish, or preparation of the manuscript?<br><br>Did you receive funding for this work?	The author(s) received no specific funding for this work.
**Competing Interests**<br><br>Use the instructions below to enter a competing interest statement for this submission. On behalf of all authors, disclose any competing interests that could be perceived to bias this work—acknowledging all financial support and any other relevant financial or non-financial competing interests.<br><br>This statement is required for submission	The authors have declared that no competing interests exist

Powered by Editorial Manager® and ProduXion Manager® from Aries Systems Corporation

and **will appear in the published article** if the submission is accepted. Please make sure it is accurate and that any funding sources listed in your Funding Information later in the submission form are also declared in your Financial Disclosure statement.<br><br>View published research articles from *PLOS ONE* for specific examples.<br><br>**NO authors have competing interests**<br><br>Enter: *The authors have declared that no competing interests exist.*<br><br>**Authors with competing interests**<br><br>Enter competing interest details beginning with this statement:<br><br>*I have read the journal's policy and the authors of this manuscript have the following competing interests: [insert competing interests here]*<br><br>* typeset	
**Ethics Statement**<br><br>Enter an ethics statement for this submission. This statement is required if the study involved:<br><br>• Human participants<br>• Human specimens or tissue<br>• Vertebrate animals or cephalopods<br>• Vertebrate embryos or tissues<br>• Field research<br><br>Write "N/A" if the submission does not require an ethics statement.<br><br>General guidance is provided below. Consult the submission guidelines for detailed instructions. **Make sure that all information entered here is included in the Methods section of the manuscript.**	This study has been approved by the Ethics Committee of the Southern Theatre General Hospital of the Chinese People's Liberation Army, with the approval number: NZLLKZ2025013

Powered by Editorial Manager® and ProduXion Manager® from Aries Systems Corporation

**Format for specific study types**<br><br>**Human Subject Research (involving human participants and/or tissue)**<br>• Give the name of the institutional review board or ethics committee that approved the study<br>• Include the approval number and/or a statement indicating approval of this research<br>• Indicate the form of consent obtained (written/oral) or the reason that consent was not obtained (e.g. the data were analyzed anonymously)<br><br>**Animal Research (involving vertebrate animals, embryos or tissues)**<br>• Provide the name of the Institutional Animal Care and Use Committee (IACUC) or other relevant ethics board that reviewed the study protocol, and indicate whether they approved this research or granted a formal waiver of ethical approval<br>• Include an approval number if one was obtained<br>• If the study involved *non-human primates*, add *additional details* about animal welfare and steps taken to ameliorate suffering<br>• If anesthesia, euthanasia, or any kind of animal sacrifice is part of the study, include briefly which substances and/or methods were applied<br><br>**Field Research**<br><br>Include the following details if this study involves the collection of plant, animal, or other materials from a natural setting:<br>• Field permit number<br>• Name of the institution or relevant body that granted permission	
**Data Availability**<br><br>Authors are required to make the data underlying their research findings fully available, without restriction.<br><br>If the manuscript reports pilot or preliminary results, the data underlying those results must be made available at the time of publication. A Data Availability	N/A - No results are reported

Powered by Editorial Manager® and ProduXion Manager® from Aries Systems Corporation

Statement describing where the data can be found is required at submission. Your answers to this question constitute the Data Availability Statement and will be published in the article, if accepted. When legal or ethical requirements prohibit public data sharing, authors must indicate how researchers may obtain access to the data. See the PLOS Data Policy and FAQ for detailed information.<br><br>**Important:** Stating 'data available on request from the author' is not sufficient. If your data are only available upon request, select 'No' for the first question and explain your situation in the text box.<br><br>For protocols without pilot or preliminary data, authors are strongly encouraged to state how they plan to share research data from their study when it is completed or published.<br><br>If your study includes pilot data, will they be made fully available without restriction?	
**Describe where the data may be found in full sentences. If you are copying our sample text, replace any instances of XXX with the appropriate details.**<br><br>• If **no pilot data are reported** in the manuscript, please enter the following: *No datasets were generated or analysed during the current study. All relevant data from this study will be made available upon study completion.*<br>• If the manuscript reports pilot data **held in a public repository**, include URLs, accession numbers or DOIs. If this information will only be available after acceptance, indicate this by ticking the box below. For example: *All XXX files are available from the XXX database (accession number(s) XXX, XXX.).*<br>• If the manuscript reports pilot data contained **within the manuscript and/or**	No datasets were generated or analysed during the current study. All relevant data from this study will be made available upon study completion.

**Supporting Information files**, enter the following:<br>*All relevant data are within the manuscript and its Supporting Information files.*<br>• If neither of these applies but you are able to provide **details of access elsewhere**, with or without limitations, please do so. For example:<br><br>*Data cannot be shared publicly because of [XXX]. Data are available from the XXX Institutional Data Access / Ethics Committee (contact via XXX) for researchers who meet the criteria for access to confidential data.*<br><br>*The data underlying the results presented in the study are available from (include the name of the third party and contact information or URL).*<br>• This text is appropriate if the data are owned by a third party and authors do not have permission to share the data.<br><br>For study protocols with data management or sharing plans, authors are encouraged to briefly describe in their Data Availability Statement how data they generate will be made accessible when the study is completed. For example:<br>• Deidentified research data will be made publicly available when the study is completed and published.<br><br>* typeset	
Additional data availability information:	

Powered by Editorial Manager® and ProduXion Manager® from Aries Systems Corporation

Manuscript Click here to access/download;Manuscript;renamed_bd9aa.docx

Dynamic Assessment of Renal Resistive Index Using Point-of-Care Ultrasonography for Early Detection of Sepsis-Induced Acute Kidney Injury: A Multicenter Clinical Trial Protocol

Runing title: Early Predictive Value of Renal Resistive Index for Sepsis-associated Acute Kidney Injury

Zongfang Ren[1#] LinglingWang[3#] JinxiYue[1] Nimei Xu[4] MingWu[5*] Zhifeng Liu[2,3*]

1. The Department of Critical Care Medicine of the Second Affiliated Hospital of Kunming Medical University,Kumming 650000,China.

2. The First Clinical College of Southern Medical University

3. Department of Medical Critical Care Medicine, General Hospital of Southern Theatre Command of Peoples Liberation Army, Guangzhou 510010, China.

4. Department of Critical Care Medicine, Jiangmen Central Hospital of Guangdong Province, Jiangmen 529000, China.

5. Department of Critical Care Medicine, Shenzhen Guangming District People's Hospital, Shenzhen 518107, China.

#These authors contributed equally to this work.

***Corresponding authors:**

Dr. Zhi-feng Liu, Department of Medical Critical Care Medicine, General Hospital of Southern Theatre Command of People’s Liberation Army, Guangzhou 510010, China.

Tel: (86)20-8868-6343; E-mail: Zhifengliu7797@163.com

Dr. Ming Wu, MD, Department of Critical Care Medicine, Shenzhen Guangming District People's Hospital, Shenzhen 518107, China. Email: boshiyy@126.com.

Abstract

Background

Acute kidney injury (AKI) is a common and serious complication of sepsis, significantly increasing both mortality risk and healthcare costs. Early prediction and diagnosis of sepsis-associated AKI (SA-AKI) remain challenging due to the delayed detection of existing renal injury markers. Although research suggests that the renal resistive index (RRI) shows promise for earlier prediction of AKI, large-scale, multicenter clinical studies to support this are currently lacking.

Methods

This study is a multicenter, open-label, non-blinded clinical trial enrolling 238 patients diagnosed with sepsis according to the Sepsis 3.0 criteria. The renal resistive index (RRI) was measured using bedside ultrasound within 2 hours of diagnosis and subsequently every 8 hours during their individualized standard treatment. Concurrently, blood samples were collected to quantify creatinine and cystatin C, and urine samples were obtained to detect kidney injury markers, including TIMP-2/IGFBP-7. The primary endpoint is to determine the threshold and accuracy of the renal resistive index in predicting sepsis-associated acute kidney injury (SA-AKI). Secondary endpoints include examining the dynamic trend of the RRI, assessing the RRI's predictive value for 28-day mortality in sepsis, and evaluating the correlation between the RRI and creatinine, cystatin C, and urinary TIMP-2/IGFBP-7 levels.

Discussion

This study assesses the accuracy and feasibility of using bedside ultrasonography

to monitor the Renal Resistive Index (RRI) for the early detection of sepsis-associated acute kidney injury (SA-AKI). Early identification of SA-AKI is crucial for improving clinical outcomes in sepsis, making this approach highly relevant for timely intervention.

Ethics and Dissemination

This study received ethical approval from the Ethics Committee of the initiator's institution. Study results will be initially reported to the collaborating investigators. Following review and consensus by the Trial Steering Committee, the research report will be drafted by the initiating investigator and disseminated through peer-reviewed publications and presentations at scientific conferences.

Trial registration

Chinese Clinical Trial Registry (ChiCTR2400082420), Registered on March 28, 2024.

Key words: Acute Kidney Injury, Sepsis, Renal Blood Flow Index, Clinical Trial，Ultrasonography

Introduction

Sepsis, defined as a life-threatening organ dysfunction caused by a dysregulated host response to infection, is frequently complicated by acute kidney injury (AKI)[1, 2]. This is the most prevalent complication of sepsis, with incidence rates ranging from 45% to 70%. Sepsis-associated AKI (SA-AKI) is a critical determinant of high mortality rates and accounts for substantial medical expenses[3, 4]. Research has consistently demonstrated that the development of SA-AKI is associated with a worse prognosis, including longer lengths of stay in intensive care units (ICUs) and hospitals. Furthermore, it is a major contributor to long-term functional impairment and reduced quality of life among sepsis survivors[5, 6]. Despite its significant impact, the absence of effective methods for the early prediction and identification of AKI poses a major challenge to clinicians in the current clinical setting and is a principal reason why AKI often fails to receive timely intervention.

Current understanding suggests that the main pathogenic mechanisms underlying SA-AKI involve endothelial cell injury within renal tissue, coupled with the activation of leukocytes and platelets to form microthrombi, ultimately leading to renal microcirculation disorders[7, 8]. An increase in renal vascular resistance is posited to be a crucial hemodynamic factor implicated in SA-AKI[9, 10]. While serum creatinine and urine output are clinical parameters widely used to evaluate renal function, they are indirect indicators reflecting physiological processes and typically manifest abnormalities only in the late stages of AKI, exhibiting relatively low sensitivity.

Therefore, exploring alternative detection methods capable of earlier prediction and assessment of SA-AKI is of significant clinical importance.

The renal resistive index (RRI), an indicator derived from Doppler ultrasound technology by measuring the blood flow spectrum of intrarenal arteries (such as interlobar or arcuate arteries), is a non-invasive parameter used to assess renal vascular resistance and parenchymal perfusion status[11-13]. As a non-invasive and repeatable bedside monitoring parameter of renal hemodynamics, RRI has been applied in the early prediction and evaluation of AKI in other contexts, such as after severe trauma or related to COVID-19 infection[14]. However, despite its potential, there remains a notable lack of large-scale, multicenter cohort studies systematically evaluating the clinical application value of RRI specifically in sepsis-associated acute kidney injury (SA-AKI).

Study Objectives

Primary Objective

The primary objective of this study is to evaluate the predictive value of dynamic monitoring of the Renal Resistive Index (RRI) for the early development of sepsis-associated acute kidney injury (SA-AKI).

Secondary Objectives

1. To assess the feasibility and reliability of implementing serial RRI measurements in critically ill septic patients at the bedside.

2. To determine optimal RRI threshold values for SA-AKI risk stratification, utilizing receiver operating characteristic (ROC) curve analysis.

3. To investigate the correlation between RRI values and established and novel biomarkers of kidney injury (serum creatinine, cystatin C, urinary TIMP-2/IGFBP-7).

4. To evaluate the prognostic significance of RRI for predicting 28-day mortality and the need for renal replacement therapy in septic patients

Methods and analysis

This protocol was prepared in accordance with the Standard Protocol Items: Recommendations for Interventional Trials (SPIRIT) guidelines (2013 edition)[15]. Following the completion of the preliminary standardized training, the recruitment period for this study is set for October 2025 to October 2027.

Trial design

This multicenter, open-label, non-blinded clinical trial enrolled a total of 238 patients diagnosed with sepsis according to the Sepsis 3.0 criteria. The study was initiated by the General Hospital of the Southern Theater Command of the People's Liberation Army (PLA). A Data Quality Monitoring Committee (DQMC) was established to provide periodic review and evaluation of trial data from each participating center. The trial design and reporting adhere to the Consolidated Standards of Reporting Trials (CONSORT) guidelines, the Declaration of Helsinki, and the principles of Good Clinical Practice (GCP)[16].

Trial seting

It will be conducted in the Intensive Care Units (ICUs) of six large tertiary teaching hospitals across mainland China.The participating ICUs were staffed by professional critical care physicians and nurses, ensuring the provision of

standardized treatment for sepsis. Furthermore, these units were equipped with intelligent central monitoring and data acquisition systems, as well as central laboratories capable of separating, preserving, and transporting plasma to a central analysis facility.

Standardization of Ultrasound Procedures

Protocolized ultrasound examinations in this study were performed by board-certified intensive care unit (ICU) physicians who had completed rigorous competency-based training; all diagnostic data underwent independent dual-reader validation with concordant interpretation.

Inclusion criteria

Patients will be considered eligible for inclusion in this trial if they meet all of the following criteria.

1. Age between 18 and 75 years, inclusive.

2. Meeting the Sepsis 3.0 diagnostic criteria and being diagnosed within 2 hours of admission to the intensive care unit (ICU).

3. Provision of written informed consent by the patient or their legally authorized representative

Exclusion Criteria

Patients meeting any of the following criteria will be excluded from participation.

1. Advanced chronic kidney disease (CKD stages 3-5 based on Kidney Disease: Improving Global Outcomes [KDIGO] guidelines).

2. Known atherosclerotic cardiovascular disease or uncontrolled diabetes mellitus requiring ongoing insulin therapy.

3. History of poorly controlled hypertension for more than 10 years despite triple antihypertensive therapy, or chronic heart failure classified as New York Heart Association (NYHA) Class III or IV.

4. Intra-abdominal hypertension Grade III or IV (defined as intra-abdominal pressure≥21 mmHg according to the World Society of the Abdominal Compartment Syndrome criteria).

5. Patients with a clinically estimated mortality risk exceeding 90% within the next 48 hours in the ICU, as determined by the Acute Physiology and Chronic Health Evaluation (APACHE) II score.

6. Confirmed pregnancy via serum β-human chorionic gonadotropin (β-hCG) testing and ultrasound examination.

7. Patients for whom inclusion is deemed inappropriate by the principal investigator due to unresolved ethical concerns, designation for palliative care, or issues related to legal guardianship.

Criteria for Participant Withdrawal

Participants may be withdrawn from the trial for any of the following reasons. The date, reason, and procedure for withdrawal will be meticulously documented in the participant's case report form.

1. Significant non-compliance with the study protocol.

2. Voluntary request for withdrawal by the participant or their legally authorized

representative.

3. Development of a serious adverse event requiring discontinuation of study procedures.

4. Other circumstances which, in the opinion of the investigator, make continuation in the trial not in the best interest of the participant or impractical.

Informed Consent

Prior to the initiation of this trial, a comprehensive assessment of participant safety was conducted during protocol design. Bedside ultrasound, as a non-invasive procedure, is not anticipated to cause irreversible or severe harm to participants. Investigators are obligated to clearly and thoroughly explain the trial objectives, potential risks, anticipated discomforts, and corresponding management strategies to participants or their legally authorized representatives. Confirmation of full comprehension of this information must be obtained. Following the provision of explicit consent, participants or their legally authorized representatives are required to sign and date the written informed consent form, thereby formally documenting their willingness to participate in this trial.

Sample Size Estimation

This study is designed as a multicenter prospective cohort study. Based on existing literature reviews, the reported incidence of sepsis-induced acute kidney injury (AKI) is approximately 55%. The sample size was calculated using PASS 15 software (NCSS, LLC). Assuming a Renal Resistive Index (RRI) sensitivity of 92% with a margin of error of 10% for sensitivity estimation, a two-tailed significance

level (α) of 0.05, and a statistical power of 95%, the minimum sample size required for achieving adequate sensitivity was calculated as 133 cases. To satisfy the specificity criteria, a minimum of 215 cases was required. Thus, the minimum sample size for the study was established as 215 cases to ensure both adequate sensitivity and specificity. To account for an estimated participant attrition rate of 10%, the final target sample size was increased to 238 participants.

Patient and Public Involvement statement

The study not appropriate to involve patients or the public in the design, or conduct, or reporting, or dissemination plans of our research.

Study procedure

Standardized Treatment

All enrolled patients received comprehensive interventions strictly adhering to the 2021 Surviving Sepsis Campaign (SSC) guidelines for standardized management. Patients diagnosed with septic shock, according to the Sepsis 3.0 criteria, received immediate administration of early goal-directed therapy (EGDT). Continuous hemodynamic monitoring was implemented, including assessment of central venous pressure (CVP), mean arterial pressure (MAP), urine output (UO), central venous oxygen saturation ($ScvO_2$), and lactate levels. The monitoring protocol was established by a board-certified critical care physician with over five years of specialized intensive care experience.

Renal Resistive Index and Renal Function Monitoring

Following confirmation of sepsis diagnosis, all patients underwent immediate

bedside renal Doppler ultrasonography monitoring. Using a 5-2 MHz convex probe, the renal resistive index (RRI) was measured bilaterally at the interlobar or arcuate arteries.To ensure consistency among all centers and operators, the measurement was conducted at the arcuate artery located at the junction of the cortex and medulla. Measurements were performed at 8-hour intervals throughout a 7-day monitoring period, with mean values recorded from three cardiac cycles per kidney. Concurrently, daily venous blood samples were collected for quantitative analysis of serum creatinine (SCr), cystatin C (CysC), and neutrophil gelatinase-associated lipocalin (NGAL). First-morning midstream urine specimens were routinely obtained for determining the ratio of tissue inhibitor of metalloproteinase-2 (TIMP-2) to insulin-like growth factor binding protein-7 (IGFBP-7).The diagnostic criteria for AKI are presented in Table 1.

Routine Monitoring Protocol

Routine monitoring was conducted throughout the study period. This included daily assessment of hematologic parameters: white blood cell count (WBC), neutrophil percentage (NEU%), and platelet count (PLT). Coagulation parameters, including prothrombin time (PT), activated partial thromboplastin time (APTT), international normalized ratio (INR), fibrinogen (FIB), and fibrinogen degradation products (FDP), were evaluated at 24-hour intervals. Biochemical markers, such as hepatic function (alanine aminotransferase [ALT], aspartate aminotransferase [AST]), electrolytes (potassium [K], sodium [Na], chloride [Cl]), and blood glucose (Glu), were also monitored every 24 hours.

Outcomes

Primary Outcome

The primary outcome is the determination of the optimal threshold value of the renal resistive index (RRI) for predicting the early development of acute kidney injury (AKI), assessed by its corresponding sensitivity, specificity, and the area under the receiver operating characteristic curve (AUC).

Secondary Outcomes

1. Assessment of the longitudinal trajectory of RRI values throughout the disease course in septic patients.

2. Evaluation of the correlation coefficients between RRI and various biomarkers.□Serum biomarkers:creatinine (Cr) and blood urea nitrogen (BUN).□Tubular injury markers:cystatinC (CysC) and neutrophil gelatinase-associated lipocalin (NGAL).□Urinary cell cycle arrest biomarkers: the ratio of tissue inhibitor of metalloproteinase-2 (TIMP-2) to insulin-like growth factor binding protein-7 (IGFBP-7).

3. Determination of the prognostic value of RRI for 28-day clinical outcomes, including mechanical ventilation duration, requirement for renal replacement therapy, and mortality.

Data Collection and Management

Data Sources and Recording

Data for this study were acquired through the Clinical Electronic Information System (CEIS) and recorded on standardized Case Report Forms (CRFs). To ensure

data quality, two independent data reviewers were appointed at each participating center. These reviewers performed a dual-review of the CRF data to guarantee accuracy, integrity, and timeliness. All completed CRFs will be stored electronically to facilitate subsequent statistical analysis and data management.

Baseline Data Collection

Upon patient enrollment, comprehensive baseline data will be collected. These data include, but are not limited to, demographic characteristics (age, sex), history of comorbid conditions, primary diagnosis, and relevant severity scoring metrics such as the Acute Physiology and Chronic Health Evaluation II (APACHE II) and the Sequential Organ Failure Assessment (SOFA). Additionally, details regarding the site of infection and identified pathogen information will be recorded.

Dynamic Monitoring Data Collection

The following dynamic monitoring data will be collected and documented on days 1, 2, 3, 5, and 7 following patient enrollment:

Vital signs: Heart rate, blood pressure, respiratory rate, and body temperature.

Laboratory testing: White blood cell count (WBC), neutrophil count, lymphocyte count, platelet count (PLT), procalcitonin (PCT), C-reactive protein (CRP), and interleukin-6 (IL-6).

Biochemical parameters: Hepatic function (alanine aminotransferase [ALT], aspartate aminotransferase [AST]), renal function (serum creatinine [SCr], blood urea nitrogen [BUN]), oxygenation index (PaO_2/FiO_2), and lactate level.

Coagulation panel: Prothrombin time (PT), activated partial thromboplastin time

(APTT), international normalized ratio (INR), fibrinogen (FIB), and D-dimer (DD).

Special Monitoring and Fluid Management: Intra-abdominal pressure (IAP) measurements (performed every eight hours and documented on these specified days). 24-hour fluid input and output (recorded daily and documented on these specified days). Fluid input encompasses crystalloids, colloids, albumin, and blood products. Fluid output includes urine volume and other drainage volumes. The 24-hour fluid balance will subsequently be calculated.

Confidentiality

The findings of this research project may be published in medical journals. However, the confidentiality of patient information will be strictly maintained in full compliance with all applicable legal and regulatory requirements. Patient personal information will not be disclosed or used for any purpose other than that explicitly outlined in this protocol, except where mandated by law. Authorized representatives of governmental regulatory authorities, institutional ethics committees, and the sponsor may be permitted to review patient records and source data for monitoring and auditing purposes, to the extent necessary to fulfill their regulatory responsibilities, while maintaining confidentiality. It is emphasized that access to confidential information will be strictly limited to authorized personnel for legitimate purposes.

Statistical analysis

Continuous variables will be presented as the mean and standard deviation (SD) for normally distributed data, or as the median and interquartile range (IQR) for

non-normally distributed data. Categorical variables will be summarized as frequencies and percentages.To analyze the relationship between the Renal Resistive Index (RRI) and 28-day mortality, a Cox proportional hazards model will be employed, with adjustments made for potential confounding variables such as center and age. Survival probabilities will be estimated using Kaplan-Meier curves, and differences in survival between distinct RRI groups will be compared using the log-rank test.The dynamic changes in RRI over time will be analyzed using a mixed-effects model for repeated measures.This model will account for within-subject variability and the influence of fixed effects, including time points, center, and age, to assess the trajectory of RRI values.The diagnostic performance of RRI for predicting acute kidney injury (AKI) incidence will be assessed using receiver operating characteristic (ROC) curve analysis. The optimal RRI threshold will be determined using the Youden Index. The 95% confidence interval for the sensitivity and specificity associated with the optimal threshold will be calculated using the Clopper-Pearson exact method.All statistical analyses will be performed using SPSS25, A two-tailed p-value of < 0.05 will be considered statistically significant.

Handling of Missing Data

Every effort will be made during the trial to minimize the occurrence of missing data, particularly for primary and secondary outcome measures. In the event of missing values, the multiple regression imputation method will be employed to handle the missing data for the statistical analyses.

Discussion

The incidence of sepsis-associated acute kidney injury (AKI) is high. Currently, the commonly used clinical indicators for diagnosis are often delayed. Researchers have been constantly searching for biomarkers that can predict AKI at an early stage. The renal resistive index (RRI) can comprehensively reflect the alterations in renal microcirculation and is capable of predicting the onset of AKI at an early phase.However, to date, no large-scale multicenter studies have been carried out among sepsis patients. The main reason might be that critically ill patients in the intensive care unit (ICU) face difficulties in being transported to the imaging diagnosis center for RRI measurement due to their critical conditions. With the widespread application of portable bedside ultrasound in the ICU, the dynamic monitoring of RRI has emerged as a highly convenient technique. This development is conducive to the implementation of this trial.

The mortality rate among patients with sepsis complicated by acute kidney injury (AKI) remains alarmingly high. This elevated mortality can be attributed, in part, to our limited understanding of the pathophysiological mechanisms underlying sepsis-associated AKI[17-19]. Recent experimental investigations have revealed that the pathogenesis of sepsis-induced AKI is considerably more intricate than mere renal hypoperfusion resulting from reduced cardiac output and hypotension.Renal microvascular alterations induced by sepsis, such as vasoconstriction, capillary leak syndrome accompanied by tissue edema, leukocyte and platelet adhesion associated with endothelial dysfunction and/or microthrombus formation, and/or an increase in intra-abdominal pressure, may contribute to an elevation in renal vascular resistance

(RVR)[20-22].Additional research is essential to elucidate the temporal sequence of renal microvascular changes during sepsis, as well as the onset and progression of acute kidney injury (AKI). Doppler ultrasonography, in conjunction with the calculation of the resistance index, can provide insights into the extent of vascular resistance alterations. This approach is instrumental in predicting the development of persistent AKI and identifying the optimal systemic hemodynamics necessary to sustain renal perfusion.

A prospective observational study conducted by Mahmoud Marzouk et al. involving 40 patients with septic shock demonstrated that the renal resistive index (RRI) could serve as an early predictor of acute kidney injury (AKI). Continuous and dynamic monitoring of RRI may play a critical role in guiding the management of septic shock[13]. Additionally, a clinical study performed by Roberta Barone et al. with 148 participants revealed that RRI is independently associated with renal dysfunction in patients undergoing coronary angiography[23]. RRI provides a more precise reflection of renal pathophysiological alterations, hemodynamic status, and functional reserve[24]. In this trial, we aim to broaden the scope of our investigation by conducting a multicenter, prospective cohort study. Through dynamic RRI monitoring and concurrent detection of both conventional and novel kidney injury biomarkers, we seek to identify optimal indicators and assessment techniques for the early prediction of sepsis-related AKI, thereby offering valuable insights and practical guidance for clinicians.

Trial Status

Recruitment for this trial commenced on October, 2025, and is anticipated to conclude by October, 2027.

Author contributions

ZFL and MW conceived of and designed the study. JXY and NMX Standardization of Ultrasound Training and Treatment Procedures, ZFR and LLW were responsible for literature retrieval and drafted the manuscript. ZFL and MW supervised the project and critically reviewed the final version of the manuscript. All authors have read and approved the final manuscript and were fully accountable for ensuring integrity and accuracy.

Funding

This research received no specific grant from any funding agency in the public, commercial, or not-for-profit sectors. This study was entirely self-funded by the research team, utilizing general institutional resources without dedicated external financial support.

Competing interests

The authors declare that they have no competing interests.

Consent for publication

Not applicable.

Acknowledgements

No acknowledgement

Reference

1. Dellinger, R.P., et al., *Surviving sepsis campaign: international guidelines for management of severe sepsis and septic shock: 2012.* Crit Care Med, 2013. **41**(2): p. 580-637.
2. Singer, M., et al., *The Third International Consensus Definitions for Sepsis and Septic Shock (Sepsis-3).* JAMA, 2016. **315**(8): p. 801-10.
3. Bougle, A. and J. Duranteau, *Pathophysiology of sepsis-induced acute kidney injury: the role of global renal blood flow and renal vascular resistance.* Contrib Nephrol, 2011. **174**: p. 89-97.
4. Zarbock, A., et al., *Sepsis-associated acute kidney injury: consensus report of the 28th Acute Disease Quality Initiative workgroup.* Nat Rev Nephrol, 2023. **19**(6): p. 401-417.
5. Fani, F., et al., *Recent advances in the pathogenetic mechanisms of sepsis-associated acute kidney injury.* J Nephrol, 2018. **31**(3): p. 351-359.
6. Peerapornratana, S., et al., *Acute kidney injury from sepsis: current concepts, epidemiology, pathophysiology, prevention and treatment.* Kidney Int, 2019. **96**(5): p. 1083-1099.
7. Katayama, S., et al., *Markers of acute kidney injury in patients with sepsis: the role of soluble thrombomodulin.* Crit Care, 2017. **21**(1): p. 229.
8. Ronco, C., R. Bellomo, and J.A. Kellum, *Acute kidney injury.* Lancet, 2019. **394**(10212): p. 1949-1964.
9. Azzouz, S., et al., *The role of renal resistive index as a prognostic tool in kidney transplantation: a systematic review.* Nephrol Dial Transplant, 2022. **37**(8): p. 1552-1565.
10. Ferreira, G.S., et al., *The Role of Biomarkers in Diagnosis of Sepsis and Acute Kidney Injury.* Biomedicines, 2024. **12**(5).
11. Darmon, M., et al., *Diagnostic accuracy of Doppler renal resistive index for reversibility of acute kidney injury in critically ill patients.* Intensive Care Med, 2011. **37**(1): p. 68-76.
12. K, C.T., S.K. Das, and M.S. Shetty, *Renal Resistive Index: Revisited.* Cureus, 2023. **15**(3): p. e36091.
13. Marzouk, M., et al., *Renal resistance index during septic shock.* Tunis Med, 2025. **103**(2): p. 270-275.
14. Giustiniano, E., et al., *Renal Resistive Index: Not Only for Kidney Clinics.* J Ultrasound Med, 2022. **41**(4): p. 1027-1030.
15. Chan, A.W., et al., *SPIRIT 2013 explanation and elaboration: guidance for protocols of clinical trials.* BMJ, 2013. **346**: p. e7586.
16. Butcher, N.J., et al., *Guidelines for Reporting Outcomes in Trial Reports: The CONSORT-Outcomes 2022 Extension.* JAMA, 2022. **328**(22): p. 2252-2264.
17. Cecconi, M., et al., *Sepsis and septic shock.* Lancet, 2018. **392**(10141): p. 75-87.
18. Labib, A., *Sepsis Care Pathway 2019.* Qatar Med J, 2019. **2019**(2): p. 4.
19. van Vught, L.A., et al., *Incidence, Risk Factors, and Attributable Mortality of Secondary Infections in the Intensive Care Unit After Admission for Sepsis.* JAMA, 2016. **315**(14): p. 1469-79.
20. Briguori, C. and G. Condorelli, *Hydration in contrast-induced acute kidney injury.* Lancet, 2014. **383**(9931): p. 1786-8.
21. Radermacher, J., et al., *Use of Doppler ultrasonography to predict the outcome of therapy for renal-artery stenosis.* N Engl J Med, 2001. **344**(6): p. 410-7.
22. Molema, G., et al., *Renal microvascular endothelial cell responses in sepsis-induced acute kidney injury.* Nat Rev Nephrol, 2022. **18**(2): p. 95-112.

23. Barone, R., et al., *Renal resistance index independently predicts worsening of renal function after coronary angiography.* Int J Cardiovasc Imaging, 2023. **39**(1): p. 43-50.

24. Cuttone, G., et al., *Exploring the Utility of Renal Resistive Index in Critical Care: Insights into ARDS and Cardiac Failure.* Biomedicines, 2025. **13**(2).

Figure1

Click here to access/download;Figure;Figure1.pdf

Figure1

	STUDY PERIOD										
	Enrolment		Post-enrolment							Close-out	
Time point (hours/in days）	Screening (T0-12h)	Baseline (0-24h-T0)	Day1 (T0+24)	Day 2	Day 3	Day 4	Day 5	Day 6	Day 7	Follow-up Discharge of ICU	Follow-up D28
Eligibility	√	√									
Informed concent		√									
Demograhics		√									
Medical history		√									
Vital signs		√									
RRI		√	Q8h	Q8h	Q8h	Q8h	Q8h	Q8h	Q8h		
Blood sample		√	√	√	√		√		√		
Biobank blood sample		√	√	√	√		√		√		
Local laboratory test (normal clinical care)		√	√	√	√		√		√		
Midstream urine (TIMP-2,IGFBP-7)		√	√	√	√		√		√		
PaO_2/FiO_2		√	√	√	√		√		√		
White blood cell count (WBC)		√	√	√	√		√		√		
Neutrophil count (Neu)		√	√	√	√		√		√		
Lymphocyte count		√	√	√	√		√		√		
Platelet count (PLT),		√	√	√	√		√		√		
Procalcitonin (PCT)		√	√	√	√		√		√		
C-reactive-protein (CRP)		√	√	√	√		√		√		
Interleukin-6 (IL-6)		√	√	√	√		√		√		
Liver function test (Bilirubin,AST,ALT)		√	√	√	√		√		√		
Renal function test(Crea,CysC,NGAL)		√	√	√	√		√		√		
Coagulation panel (PT,APTT,Fib,DD,INR)		√	√	√	√		√		√		
Intra-abdominal pressure (IAP)		√	√	√	√		√		√		
Fluid inputt		√	√	√	√	√	√	√	√		
Fluid output		√	√	√	√	√	√	√	√		
SOFA		√	√	√	√	√	√	√	√		
APACHE II		√	√	√	√	√	√	√	√		

Figure2

Click here to access/download;Figure;Figure1.jpg

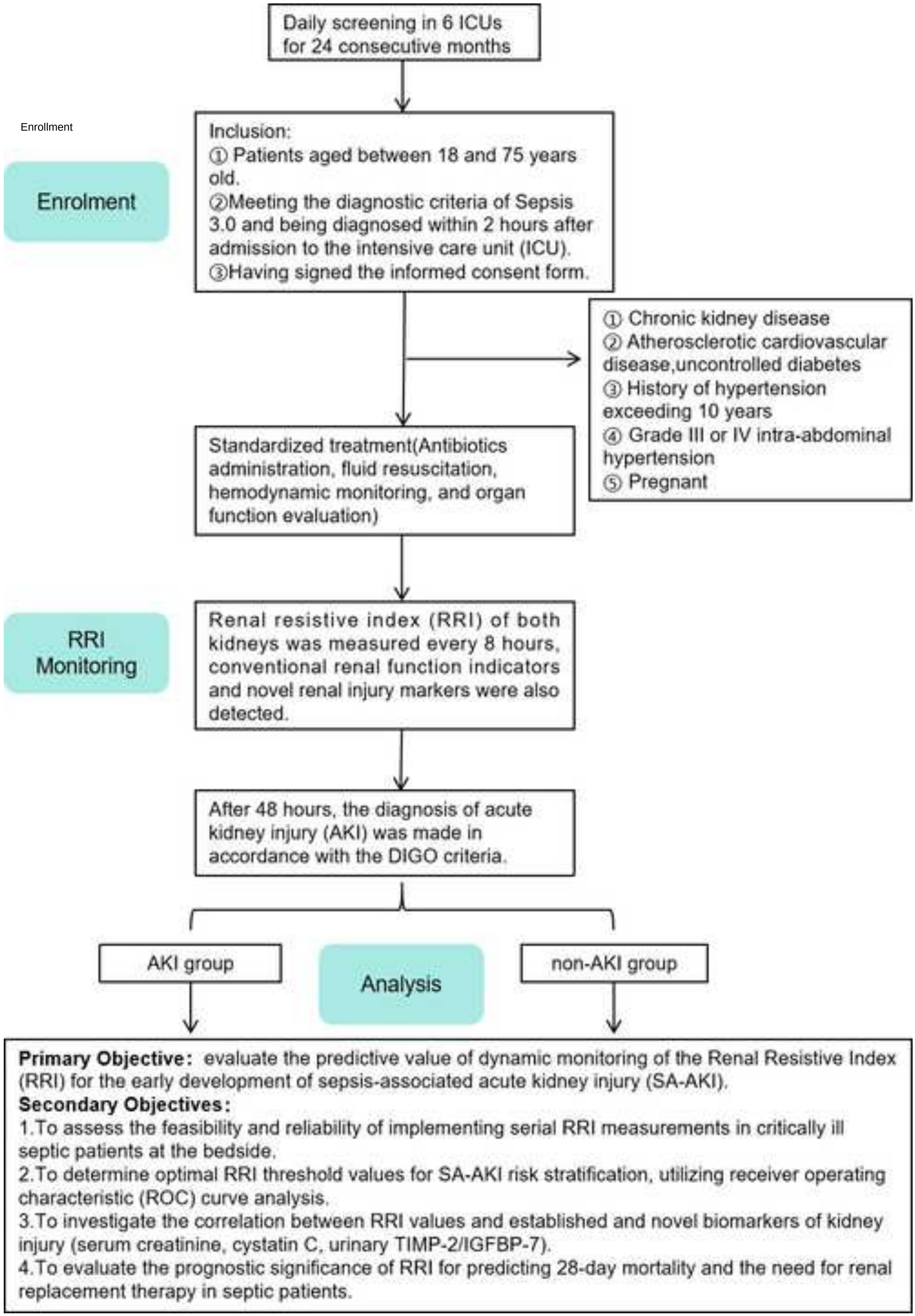

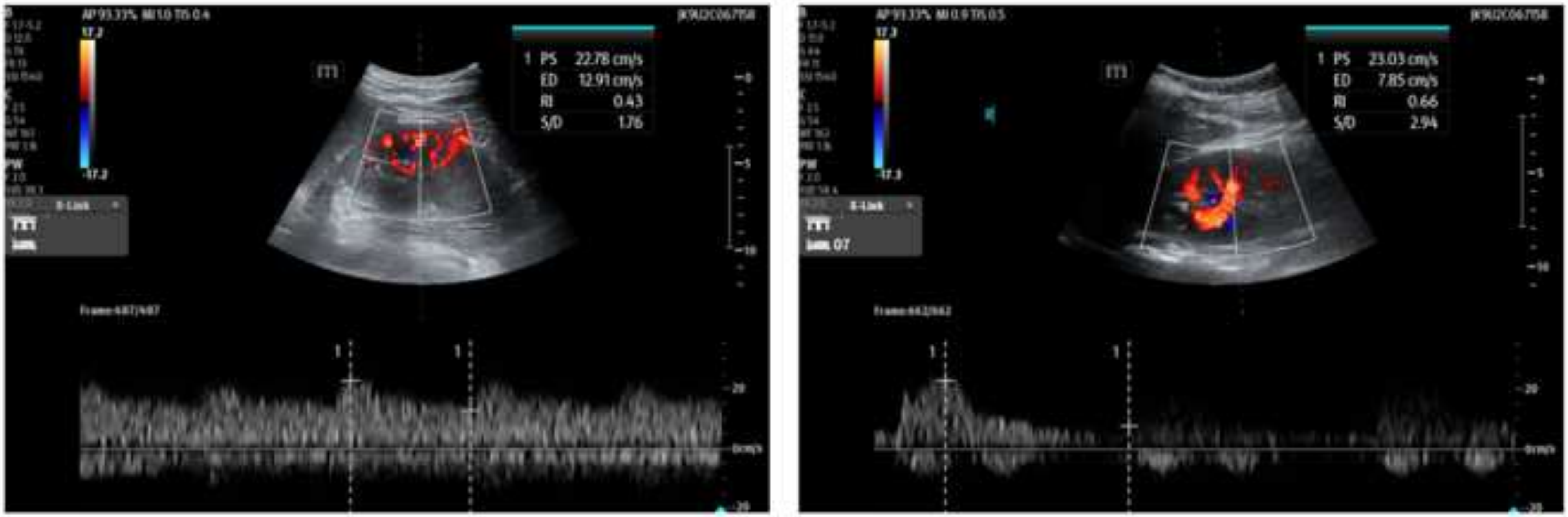

Figure 2. Evaluation of the renal resistive index using Doppler ultrasound. The transducer is placed in an interlobar artery, and the spectral Doppler examines the peak systolic and end-diastolic velocities

Are you going to measure doppler waveforms from interlobar artery or arcunate artery ?

AKI Diagnostic Critical	
Stage	Serum Creatinine and Urine Output
I	Increase in SCr of 1.5–1.9 times baseline or ≥0.3 mg/dL .<br>Urine output <0.5 mL/kg/hr for 6–12 hours.
II	Increase in SCr of 2.0–2.9 times baseline.<br>Urine output <0.5 mL/kg/hr for ≥12 hours.
III	Increase in SCr of ≥3.0 times baseline or SCr ≥4.0 mg/dL (≥354 μmol/L), or initiation of renal replacement therapy (RRT).<br>Urine output <0.3 mL/kg/hr for ≥24 hours, or anuria for ≥12 hours

Table 1. Acute kidney injury (AKI) diagnostic critical

Click here to access/download
Supporting Information
SPIRIT-Checklist.doc

Click here to access/download
Supporting Information
Clinical Study Protocol on the Renal Blood Flow Re.pdf

Click here to access/download
Supporting Information
renamed_945d0.pdf

Click here to access/download
Supporting Information
renamed_0adf6.pdf
