# Supplementary material for: Dynamic assessment of renal resistive index using point-of-care ultrasonography for early detection of sepsis-induced acute kidney injury: A multicenter clinical trial protocol
Source: PLoS One. 2026 Oct 29;21(10):e0359982. doi: 10.1371/journal.pone.0359982 (PMC13718935; doi:10.1371/journal.pone.0359982)
Supplement: Supplementary file 2 [file pone.0359982.s002.docx]

**General assessment**

This manuscript outlines the protocol for a multicentre study that evaluates the predictive value of the renal resistive index (RRI), as measured by point-of-care ultrasonography, for the early detection of sepsis-associated acute kidney injury (SA-AKI). This is a clinically relevant topic, as the early detection of AKI in septic patients remains a major challenge in critical care medicine. Using a non-invasive bedside parameter such as the RRI could potentially improve the early stratification of risk and guide clinical decision-making.

Strengths of the proposed study include the multicentre design, dynamic RRI monitoring and simultaneous measurement of several kidney injury biomarkers. However, several important methodological and conceptual issues need to be clarified before the protocol can be adequately evaluated. In particular, further clarification is required on the description of the study design, the statistical strategy, the definition of outcomes and the control of potential confounders influencing RRI.

Below, I provide major and minor comments intended to improve the clarity and methodological rigour of the manuscript.

**Major comments**

*1) Clarification of the study design*

In one section of the manuscript, the study is described as a 'multicentre, open-label, non-blinded clinical trial', whereas in another section, it is described as a 'multicentre, prospective cohort study'.

However, according to the protocol description:

There is no intervention being tested.

All patients receive standard sepsis management.

There is no randomisation or comparison group.

Therefore, the study appears to be an observational prospective cohort study rather than a clinical trial. This distinction is important because the manuscript refers to the CONSORT and SPIRIT guidelines, which are primarily intended for randomised or interventional trials.

***Questions for the authors:***

Please clarify whether this study is an observational cohort study or an interventional clinical trial.

If the study is observational, the terminology and reporting framework should be revised accordingly.

*2) Definition of the primary outcome and timing of AKI prediction*

While the primary objective is to determine the predictive value of RRI for the early development of SA-AKI, the manuscript does not clearly specify the time window used to define 'early' AKI prediction.

It is unclear whether RRI measured at baseline is intended to predict AKI occurring within:

24 hours/48 hours /72 hours? or during the 7-day monitoring period.

The predictive performance of RRI (e.g. ROC analysis) depends heavily on the defined prediction window.

***Questions for the authors:***

What time frame was used to define AKI prediction?

Is the primary analysis based on baseline RRI predicting AKI within a specific timeframe (e.g. 48 hours)?

Please clarify how the timing of AKI onset will be incorporated into the statistical analysis.

*3) Potential confounding factors affecting RRI*

Numerous systemic and haemodynamic factors influence the renal resistive index, including:

mean arterial pressure/use and dosage of vasopressors/Mechanical ventilation/Positive end-expiratory pressure (PEEP)/Intra-abdominal pressure/Vascular stiffness and age/Fluid balance

Although intra-abdominal pressure and fluid balance are recorded, it is unclear whether these variables will be adjusted for in multivariable analyses.

Without appropriate adjustment, changes in RRI may reflect systemic haemodynamic alterations rather than renal microcirculatory injury.

***Questions for the authors:***

Which haemodynamic variables will be included in the multivariable models?

Will potential confounders such as vasopressor dose, mean arterial pressure, mechanical ventilation status and PEEP be considered?

*4) Reproducibility and inter-observer variability of RRI measurements*

RRI measurements are known to be operator-dependent. The manuscript states that ultrasound examinations will be performed by trained ICU physicians and that the data will undergo dual-reader validation. However, no information is provided regarding:

- assessment of inter-observer variability

- intra-observer reproducibility

Whether image acquisition protocols will be standardised across centres is also unclear.

This is particularly important in a multicentre study involving repeated measurements.

***Questions for the authors:***

Will reproducibility analyses (e.g. the intraclass correlation coefficient) be performed?

How many operators will perform RRI measurements in each centre?

Will image recordings be stored and centrally reviewed?

*5)Feasibility of repeated RRI measurements*

The protocol proposes RRI measurements every 8 hours for 7 days, which corresponds to approximately 21 measurements per patient.

Given the planned sample size of 238 patients, this represents several thousand Doppler examinations. The feasibility of such a monitoring protocol in busy ICUs should be addressed.

***Questions to the authors***

Was this monitoring frequency tested in a pilot study?

What strategies will be implemented to ensure protocol adherence across centers?

How will missing RRI measurements be handled?

*6) Sample size calculation*

The sample size calculation assumes a sensitivity of 92% for RRI, which is relatively high compared to that reported in previous studies on RRI for predicting AKI.

However, the manuscript does not provide sufficient justification for this assumption.

***Questions for the authors:***

Which study supports the assumption of 92% sensitivity?

Would it be more appropriate to base the sample size calculation on the expected area under the curve (AUC) values for the receiver operating characteristic (ROC) analysis rather than sensitivity alone?

*7) Definition of the RRI exposure variable:*

It is unclear which RRI parameter will be used in the main analyses.

Baseline RRI/maximum RRI during follow-up/mean RRI/Change in RRI over time.

Given the repeated measurements planned in the study, this issue should be clarified.

***Question for the authors:***

Which RRI variable will be used as the primary predictor in the main analysis?

*8) Risk of statistical overfitting*

The estimated AKI incidence is approximately 55%, which would result in roughly 130 AKI events.

If multiple covariates are included in multivariable models, there is a potential risk of overfitting.

***Question to the authors***

How many variables are planned for inclusion in the final multivariable models?

Was the events-per-variable rule considered during model development?

**Minor comments**

There are several minor language and typographical errors throughout the manuscript that should be corrected. For example:

'Running title' should be corrected to 'Running title'.

'Trial setting' should be corrected to 'Trial setting'.

'Informed consent' should be corrected to 'informed consent'.

Some references are relatively outdated (e.g. the Surviving Sepsis Campaign, 2012). Where appropriate, more recent guidelines (e.g. SSC 2021) should be cited.

The term 'renal blood flow index' is not commonly used in the literature. It would therefore be more appropriate to use 'renal resistive index'.

The manuscript sometimes refers to NGAL as a biomarker, yet in other sections, it is not listed among the planned measurements. Please ensure consistency across sections.

The description of the statistical software (SPSS 25) could be accompanied by the name and location of the manufacturer.

In figure 2, Please correct KDIGO

**Conclusion**

This protocol addresses an important clinical question regarding the potential role of the renal resistive index as an early predictor of sepsis-associated acute kidney injury. The multicenter design and the dynamic monitoring strategy represent strengths of the proposed study.

However, several methodological aspects require clarification, particularly regarding the study design, outcome definition, adjustment for confounders, measurement reproducibility, and statistical analysis plan. Addressing these issues would substantially improve the clarity and scientific rigor of the protocol. Therefore, I recommend **major revision** before the manuscript can be considered for publication.
