# Supplementary material for: Dynamic assessment of renal resistive index using point-of-care ultrasonography for early detection of sepsis-induced acute kidney injury: A multicenter clinical trial protocol
Source: PLoS One. 2026 Oct 29;21(10):e0359982. doi: 10.1371/journal.pone.0359982 (PMC13718935; doi:10.1371/journal.pone.0359982)
Supplement: Supplementary file 3 [file pone.0359982.s004.docx]

Response to Reviewers

**Reviewer #1:** Very well-written protocol using SPIRIT checklist. I am interested in learning the results from your study, this can improve clinical practice with common use of bedside ultrasound. There are two typos in the image section - Inputt, Enrolment. Otherwise, everything looks good.

**Answer:** Thank you for your comments. We have corrected Table 2 accordingly, with the changes indicated in red font.

**Reviewer #2:** Reviewer Comments to the Authors

**Major Comments**
1. Clarification of the study design
The manuscript is described as a “multicenter clinical trial protocol.” Based on the methods presented, the study appears to be a prospective observational cohort rather than an interventional clinical trial. The authors should clarify the study design and ensure that the terminology used throughout the manuscript accurately reflects the methodological framework.

**Answer:** Thank you for your comments. The study is a prospective observational cohort study, not an interventional trial. All relevant sections have been revised for accuracy, and the changes are highlighted in red font throughout the manuscript.
2. Rationale for the 7-day monitoring period
**Answer:** We appreciate your comment. Sepsis-associated acute kidney injury is generally defined as AKI occurring within 7 days of sepsis onset, a timeframe based primarily on clinical observations and evidence-based standards. AKI developing after 7 days is usually related to other factors, which is why we chose a 7-day monitoring period.

**Minor Comments**
1. Potential confounding factors affecting RRI
The renal resistive index can be influenced by several systemic and hemodynamic factors in critically ill patients. In addition to mean arterial pressure and heart rate, intra-abdominal pressure, as well as variables such as vasopressor use, mechanical ventilation parameters (e.g.PEEP), and fluid status may also significantly affect RRI values. Clarification is needed regarding whether these factors will be recorded and adjusted for in the statistical analysis.

**Answer:** Thank you for your valuable comments. Our study also includes parameters such as intra-abdominal pressure, fluid intake, and fluid output. In addition, we have updated the Figures to include mean arterial pressure, heart rate, vasoactive drug dose and duration, mechanical ventilation duration, and PEEP. These parameters will be incorporated into the statistical analysis to provide a more comprehensive and objective evaluation of the study results. All added content has been highlighted in red font.
2. Definition of baseline serum creatinine
The manuscript states that AKI will be diagnosed according to KDIGO 2012 criteria using serum creatinine and urine output. However, the method used to determine baseline serum creatinine is not clearly described, particularly in patients without recent laboratory data prior to admission. The authors should clarify how baseline creatinine will be defined.

**Answer:** Thank you for important comment. Baseline serum creatinine will be defined as follows:

① Patients with recent laboratory data (within 3 months prior to admission): the most recent pre-admission serum creatinine value will be used.

② Patients without recent measurements: baseline creatinine will be estimated using the Modification of Diet in Renal Disease (MDRD) equation, assuming a normal baseline eGFR of 75 mL/min/1.73 m², in accordance with KDIGO 2012 recommendations. This clarification has been added to the Methods section (changes highlighted in red font ).
3. Clinical implications of RRI monitoring
While the study focuses on the predictive value of RRI for SA-AKI, the manuscript could further clarify how early identification of elevated RRI might influence clinical decision-making in septic patients.

**Answer:** Thank you for this valuable comment. Early detection of elevated RRI in septic patients may allow clinicians to anticipate AKI before traditional biochemical markers change, enabling timely interventions such as hemodynamic optimization, fluid management adjustments, and avoidance of nephrotoxic agents. We have added a brief clarification in the discussion section to highlight how RRI monitoring could guide early clinical decision-making and potentially improve patient outcomes (changes highlighted in red font ).

**Reviewer #3:**

**Major comments**

1. Clarification of the study design

In one section of the manuscript, the study is described as a 'multicentre, open-label, non-blinded clinical trial', whereas in another section, it is described as a 'multicentre, prospective cohort study'.However, according to the protocol description:There is no intervention being tested. All patients receive standard sepsis management.There is no randomisation or comparison group.Therefore, the study appears to be an observational prospective cohort study rather than a clinical trial. This distinction is important because the manuscript refers to the CONSORT and SPIRIT guidelines, which are primarily intended for randomised or interventional trials.

**Answer:** Thank you for highlighting this important point. We apologize for the previous inconsistency in terminology. This study is a multicentre, prospective observational cohort study with no intervention, randomization, or comparison group. Accordingly, all references to “clinical trial” have been corrected, and citations to CONSORT and SPIRIT guidelines have been replaced with the STROBE (Strengthening the Reporting of Observational Studies in Epidemiology) guidelines, which are appropriate for observational cohort studies. All changes have been highlighted in red font throughout the manuscript.

2. Questions for the authors

Please clarify whether this study is an observational cohort study or an interventional clinical trial. If the study is observational, the terminology and reporting framework should be revised accordingly.
**Answer:** Thanks your comment. This study is a prospective observational cohort study with no intervention, randomization, or comparison group. Accordingly, all terminology has been updated to reflect this study design, and reporting now follows the STROBE (Strengthening the Reporting of Observational Studies in Epidemiology) guidelines, which are appropriate for observational cohort studies. All changes have been highlighted in red font throughout the manuscript.

1) Definition of the primary outcome and timing of AKI prediction

While the primary objective is to determine the predictive value of RRI for the early development of SA-AKI, the manuscript does not clearly specify the time window used to define 'early' AKI prediction.It is unclear whether RRI measured at baseline is intended to predict AKI occurring within: 24 hours/48 hours /72 hours? or during the 7-day monitoring period.The predictive performance of RRI (e.g. ROC analysis) depends heavily on the defined prediction window.

**Answer:** Thank you the insightful comment. In the revised manuscript, we have clarified that **“early AKI” is defined as AKI occurring within 48 hours after sepsis diagnosis (early SA-AKI)**. Accordingly, **the renal resistive index (RRI) measured at baseline is intended to predict AKI within this 48-hour window**.This 48-hour prediction window is based on current literature showing that early SA-AKI typically develops within the first 48 hours of sepsis onset, while late SA-AKI occurs between 48 hours and 7 days (Zarbock et al., 2023). **All ROC analyses and other evaluations of RRI predictive performance are based on AKI events occurring within this 48-hour period**, ensuring clinical relevance and accuracy.These clarifications have been **added to the Methods section in red font** to highlight the correspondence between RRI measurement and the defined early AKI prediction window.

****Reference****
Zarbock A, Nadim MK, Pickkers P, et al. Sepsis‑associated acute kidney injury: consensus report of the 28th Acute Disease Quality Initiative workgroup. Nat Rev Nephrol. 2023;19(6):401–417. [https://www.nature.com/articles/s41581-023-00683-3](https://www.nature.com/articles/s41581-023-00683-3?utm_source=chatgpt.com)

2) Questions for the authors:

What time frame was used to define AKI prediction? Is the primary analysis based on baseline RRI predicting AKI within a specific timeframe (e.g. 48 hours)? Please clarify how the timing of AKI onset will be incorporated into the statistical analysis.

**Answer:** We sincerely thank you for your insightful comment. In this study, “early AKI” is defined as AKI occurring within 48 hours after sepsis diagnosis (early SA-AKI). The primary analysis is based on Mean RRI to predict the occurrence of AKI within 48 hours. In the statistical analysis, the timing of AKI onset is explicitly recorded, and occurrence of AKI within 48 hours is used as the primary outcome. Therefore, ROC analysis and other evaluations of RRI predictive performance are based on AKI events occurring within this 48-hour period, ensuring clinical relevance and accuracy. These clarifications have been added to the methods section in red font.

3) Potential confounding factors affecting RRI

Numerous systemic and haemodynamic factors influence the renal resistive index, including: mean arterial pressure/use and dosage of vasopressors/Mechanical ventilation/Positive end-expiratory pressure (PEEP)/Intra-abdominal pressure/Vascular stiffness and age/Fluid balance Although intra-abdominal pressure and fluid balance are recorded, it is unclear whether these variables will be adjusted for in multivariable analyses. Without appropriate adjustment, changes in RRI may reflect systemic haemodynamic alterations rather than renal microcirculatory injury.

**Answer:** Thank you for this important comment. We acknowledge that multiple systemic and hemodynamic factors—including MAP, vasopressor use and dosage, mechanical ventilation, PEEP, intra-abdominal pressure, vascular stiffness, age, and fluid balance—can influence RRI. In our study, we have added the recording of the suggested factors (e.g., MAP, PEEP, etc.), and patients with severe arterial stiffness were already excluded according to our exclusion criteria.

These variables will be incorporated as covariates in the multivariable analyses to adjust for potential confounding effects. This clarification has been added to the manuscript in red font.

**Questions for the authors:**

1) Which haemodynamic variables will be included in the multivariable models? Will potential confounders such as vasopressor dose, mean arterial pressure, mechanical ventilation status and PEEP be considered?

**Answer**: Thank you for the question. In the multivariable models, the following hemodynamic variables will be included: mean arterial pressure (MAP), vasopressor use and dosage, mechanical ventilation status, PEEP, intra-abdominal pressure, vascular stiffness, age, and fluid balance. These potential confounders will be adjusted for to ensure that changes in RRI primarily reflect renal microcirculatory alterations rather than systemic hemodynamic fluctuations. This clarification has been added to the Statistical Analysis sections in red font.

2) Reproducibility and inter-observer variability of RRI measurements RRI measurements are known to be operator-dependent. The manuscript states that ultrasound examinations will be performed by trained ICU physicians and that the data will undergo dual-reader validation. However, no information is provided regarding: assessment of inter-observer variability; intra-observer reproducibility

Whether image acquisition protocols will be standardised across centres is also unclear.This is particularly important in a multicentre study involving repeated measurements.

**Answer:** Thank you for your valuable comments. We have clarified the following in the Methods section:

**Reproducibility and inter-observer variability:** RRI measurements will be performed by trained ICU physicians. Each image will be independently assessed by two readers. We will report inter- and intra-observer reliability using Intraclass Correlation Coefficient (ICC) for continuous data and Cohen’s kappa for categorical data.

**Standardization of image acquisition:** All centers will follow a unified ultrasound protocol, including probe position, angle, and respiratory phase, to ensure comparability across repeated measurements in this multicenter study. These points have been highlighted in red font in the Methods section.

**Questions for the authors:**

1) Will reproducibility analyses (e.g. the intraclass correlation coefficient) be performed? How many operators will perform RRI measurements in each centre?

Will image recordings be stored and centrally reviewed?

**Answer**: Thank you for your comments. Reproducibility will be assessed using ICC for continuous measurements and Cohen’s kappa for categorical data. Each center will have three trained ICU physicians performing RRI measurements. All images will be digitally stored and uploaded weekly to the central team for review. Standardized ultrasound protocols will be followed across centers to ensure consistency. These clarifications have been added to manuscript and are highlighted in red font

2) Feasibility of repeated RRI measurements: The protocol proposes RRI measurements every 8 hours for 7 days, which corresponds to approximately 21 measurements per patient.Given the planned sample size of 238 patients, this represents several thousand Doppler examinations. The feasibility of such a monitoring protocol in busy ICUs should be addressed.

**Answer**: Thank you for your question. This study will be conducted in the ICUs of large tertiary hospitals. In the routine management and fluid therapy of sepsis, bedside ultrasound assessments are required to be performed repeatedly according to the Chinese Critical Care Quality Control standards. In these ICUs, senior physicians typically conduct rounds every 8 hours; therefore, we have set the RRI measurement interval to 8 hours to align with clinical workflows and to avoid interfering with routine patient care. These clarifications have been discussed in the manuscript and are highlighted in red.

**Questions to the authors**

1) Was this monitoring frequency tested in a pilot study? What strategies will be implemented to ensure protocol adherence across centers? How will missing RRI measurements be handled?

**Answer**: Thank you for your question. During the pilot phase, the proposed RRI measurement schedule of every 8 hours was tested in selected ICU beds and was confirmed not to interfere with routine patient care. Consistency across centers will be ensured through regular communication, supervision, and centralized review of image records. Any missing RRI measurements will be documented and handled using appropriate statistical methods, such as multiple imputation. If a patient has five or more missing measurements, they will be excluded from the analysis and not included in the statistical models. These clarifications have been added to manuscript highlighted in red font.

2) Sample size calculation

The sample size calculation assumes a sensitivity of 92% for RRI, which is relatively high compared to that reported in previous studies on RRI for predicting AKI.

However, the manuscript does not provide sufficient justification for this assumption.

**Answer**: Thank you for your comment regarding the assumed sensitivity of RRI in our sample size calculation. Previous studies and meta-analyses have reported that the sensitivity of RRI for predicting AKI ranges from 72% to 83%, with specificity around 79% to 82%, showing even better predictive performance in ICU patients. In our study, we assumed a sensitivity of 92% based on results from our pilot study in large tertiary hospital ICUs, considering that RRI measurements will be performed by trained physicians following standardized protocols, and repeated measurements will enhance overall early AKI prediction.

**Questions for the authors:**

1) Which study supports the assumption of 92% sensitivity? Would it be more appropriate to base the sample size calculation on the expected area under the curve (AUC) values for the receiver operating characteristic (ROC) analysis rather than sensitivity alone?

**Answer:** Thank you for your suggestion. We assumed a sensitivity of 92% for RRI based on results from our pilot study conducted in large tertiary hospital ICUs; this value is slightly higher than reported in previous literature. Sample size calculation based on the AUC reflects the predictive performance of the marker across all possible thresholds, providing a more comprehensive assessment than using sensitivity alone. We have revised the sample size calculation accordingly, and these updates have been incorporated into the manuscript and highlighted in red font.

2) Definition of the RRI exposure variable: It is unclear which RRI parameter will be used in the main analyses.Baseline RRI/maximum RRI during follow-up/mean RRI/Change in RRI over time.Given the repeated measurements planned in the study, this issue should be clarified.

**Answer:** Thank you for your comment regarding the definition of the RRI exposure variable. In our study, we plan to use both baseline RRI and the maximum RRI during the follow-up period as the primary exposure variables in the main analyses. Additionally, mean RRI over time and changes in RRI will be explored in secondary analyses to assess dynamic trends. Given the repeated measurements, all RRI values will be measured using standardized protocols, and summary measures (baseline, maximum, mean, change) will be clearly defined in the Methods section. These clarifications have been added to the manuscript and highlighted in red font.

**Question for the authors:**

1) Which RRI variable will be used as the primary predictor in the main analysis?

**Answer:** Thank you for your question. In the primary analysis of this study, Mean RRI will be used as the main predictor. The maximum RRI during follow-up, Baseline RRI, and change in RRI over time will be included as secondary analyses to evaluate dynamic trends. These clarifications have been added to the Methods section of the manuscript and are highlighted in red font.

2) Risk of statistical overfitting

The estimated AKI incidence is approximately 55%, which would result in roughly 130 AKI events. If multiple covariates are included in multivariable models, there is a potential risk of overfitting.

**Answer:** Thank you for your comment regarding the risk of overfitting in multivariable analyses. The estimated AKI incidence is approximately 55%, corresponding to roughly 130 events. To minimize overfitting, only key covariates with clear clinical relevance will be included, ensuring sufficient events per variable (EPV ≥10), and model robustness will be assessed using cross-validation or bootstrap methods. These clarifications have been added to the Methods section and are highlighted in red font.

**Question to the authors**

1) How many variables are planned for inclusion in the final multivariable models? Was the events-per-variable rule considered during model development?

**Answer:** Thank you for your comment regarding variable selection in the multivariable models. We plan to include 8–10 key covariates in the final models, with a maximum of 13 variables, all selected based on clinical relevance and prior literature. The events-per-variable (EPV ≥10) principle has been fully considered to ensure model robustness and minimize the risk of overfitting. These clarifications have been added to the manuscript and are highlighted in red font.

**Minor comments**

There are several minor language and typographical errors throughout the manuscript that should be corrected. For example:

1.'Running title' should be corrected to 'Running title'.

**Answer:** Thank you for your comment. The manuscript has been revised accordingly, and the changes are highlighted in red font.

2.'Trial setting' should be corrected to 'Trial setting'.

**Answer:** Thank you for your comment. The manuscript has been revised accordingly, and the changes are highlighted in red font.

3.'Informed consent' should be corrected to 'informed consent'.

**Answer:** Thank you for your comment. The manuscript has been revised accordingly, and the changes are highlighted in red font.

4.Some references are relatively outdated (e.g. the Surviving Sepsis Campaign, 2012).

**Answer:** Thank you for your comment. We acknowledge that some references were relatively outdated (e.g., the Surviving Sepsis Campaign, 2012). We have updated the manuscript to cite the most recent Surviving Sepsis Campaign: International Guidelines for Management of Sepsis and Septic Shock 2026 and other relevant up-to-date literature. These changes have been incorporated into the manuscript and highlighted in red font.

5.Where appropriate, more recent guidelines (e.g. SSC 2021) should be cited.

**Answer:** Thank you for your comment. Where appropriate, we have updated the references to cite the latest Surviving Sepsis Campaign 2026 (SSC 2026). These changes have been incorporated into the manuscript and are highlighted in red font. Thank you again for your valuable feedback.

6.The term 'renal resistive index' is not commonly used in the literature. It would therefore be more appropriate to use 'renal resistive index'.

**Answer:** Thank you for your suggestion. We have updated all relevant expressions in the manuscript to “renal resistive index (RRI)” and highlighted the changes in red font.

7.The manuscript sometimes refers to NGAL as a biomarker, yet in other sections, it is not listed among the planned measurements. Please ensure consistency across sections.

**Answer:** We appreciate your comment regarding NGAL. As this study does not involve NGAL, we have revised the manuscript accordingly.

8.The description of the statistical software (SPSS 25) could be accompanied by the name and location of the manufacturer.

**Answer:** Thank you for your comment. We have added the manufacturer information for the statistical software in the manuscript. Specifically, the description of SPSS 25 has been updated to “SPSS version 25 (IBM Corp., Armonk, NY, USA),” and the added parts are highlighted in red font.

9.In figure 1, Please correct KDIGO

**Answer:** Thank you for your comment. We have corrected the spelling errors in Figure1.
