# Supplementary material for: Dynamic assessment of renal resistive index using point-of-care ultrasonography for early detection of sepsis-induced acute kidney injury: A multicenter clinical trial protocol
Source: PLoS One. 2026 Oct 29;21(10):e0359982. doi: 10.1371/journal.pone.0359982 (PMC13718935; doi:10.1371/journal.pone.0359982)
Supplement: Supplementary file 4 [file pone.0359982.s005.docx]

Response to Reviewers

**Minor Revisions Required**

1. Inconsistent biomarker information between the main text and Figure 3 The main text clearly states that NGAL will not be tested in this study, whereas the timeline table in Figure 3 still contains items related to NGAL. This inconsistency may confuse readers. The authors should remove all NGAL-related entries from Figure 3.

**Answer:**Thank you for your valuable comment. We have removed all NGAL-related entries from Figure 3.

2. Uncorrected spelling error in Figure 3 The authors have revised the misspellings “Inputt” and “Enrolment”, and the typo “Informed concent” in Figure 1 has also been fixed. Nevertheless, the same spelling error remains in Figure 3: the phrase “Informed concent” is incorrect, and the standard clinical term is “Informed consent”.

**Answer:**Thank you for your valuable comment. We have corrected the spelling error in Figure 3. The corresponding revision has been made in the revised manuscript.

3. Incomplete Data Availability Statement The text only mentions that all relevant research data will be released after trial completion, without specifying the repository, storage location or access channel for the final dataset. In accordance with PLOS ONE’s open data policy, the authors should add detailed information about where the complete trial data will be archived and accessed once recruitment finishes.

**Answer:** Thank you for your valuable comment. We appreciate your suggestion regarding the Data Availability Statement. The relevant research data will be deposited in the Figshare public repository upon completion of this clinical trial and publication of the study. The dataset will be freely available through a permanent DOI, which will be included in the final published article.
