# Supplementary material for: Dynamic assessment of renal resistive index using point-of-care ultrasonography for early detection of sepsis-induced acute kidney injury: A multicenter clinical trial protocol
Source: PLoS One. 2026 Oct 29;21(10):e0359982. doi: 10.1371/journal.pone.0359982 (PMC13718935; doi:10.1371/journal.pone.0359982)
Supplement: Peer review history

# Peer review: Decision Letter 0

**DOI:** 10.1371/journal.pone.0359982.r001

**Authors:** Ramya Iyadurai

21 Mar 2026

Dear Dr. Ren,

Thank you for submitting your manuscript to PLOS ONE. After careful consideration, we feel that it has merit but does not fully meet PLOS ONE’s publication criteria as it currently stands. Therefore, we invite you to submit a revised version of the manuscript that addresses the points raised during the review process.

Please submit your revised manuscript by May 05 2026 11:59PM. If you will need more time than this to complete your revisions, please reply to this message or contact the journal office at plosone@plos.org. When you're ready to submit your revision, log on to https://www.editorialmanager.com/pone/ and select the 'Submissions Needing Revision' folder to locate your manuscript file.

- A letter that responds to each point raised by the academic editor and reviewer(s). You should upload this letter as a separate file labeled 'Response to Reviewers'.
- A marked-up copy of your manuscript that highlights changes made to the original version. You should upload this as a separate file labeled 'Revised Manuscript with Track Changes'.
- An unmarked version of your revised paper without tracked changes. You should upload this as a separate file labeled 'Manuscript'.

If you would like to make changes to your financial disclosure, please include your updated statement in your cover letter. Guidelines for resubmitting your figure files are available below the reviewer comments at the end of this letter.

If applicable, we recommend that you deposit your laboratory protocols in protocols.io to enhance the reproducibility of your results. Protocols.io assigns your protocol its own identifier (DOI) so that it can be cited independently in the future. For instructions see: https://journals.plos.org/plosone/s/submission-guidelines#loc-laboratory-protocols. Additionally, PLOS ONE offers an option for publishing peer-reviewed Lab Protocol articles, which describe protocols hosted on protocols.io. Read more information on sharing protocols at https://plos.org/protocols?utm_medium=editorial-email&utm_source=authorletters&utm_campaign=protocols.

We look forward to receiving your revised manuscript.

Kind regards,

Ramya Iyadurai

Academic Editor

PLOS One

Journal Requirements:

When submitting your revision, we need you to address these additional requirements.

1.Please ensure that your manuscript meets PLOS ONE's style requirements, including those for file naming. The PLOS ONE style templates can be found at

https://journals.plos.org/plosone/s/file?id=wjVg/PLOSOne_formatting_sample_main_body.pdf and

https://journals.plos.org/plosone/s/file?id=ba62/PLOSOne_formatting_sample_title_authors_affiliations.pdf

2. PLOS requires an ORCID iD for the corresponding author in Editorial Manager on papers submitted after December 6th, 2016. Please ensure that you have an ORCID iD and that it is validated in Editorial Manager. To do this, go to ‘Update my Information’ (in the upper left-hand corner of the main menu), and click on the Fetch/Validate link next to the ORCID field. This will take you to the ORCID site and allow you to create a new iD or authenticate a pre-existing iD in Editorial Manager.

3. Please include your tables as part of your main manuscript and remove the individual files. Please note that supplementary tables (should remain/ be uploaded) as separate "supporting information" files.

4. Please provide a complete Data Availability Statement in the submission form, ensuring you include all necessary access information or a reason for why you are unable to make your data freely accessible. If your research concerns only data provided within your submission, please write "All data are in the manuscript and/or supporting information files" as your Data Availability Statement.

5. When completing the data availability statement of the submission form, you indicated that you will make your data available on acceptance. We strongly recommend all authors decide on a data sharing plan before acceptance, as the process can be lengthy and hold up publication timelines. Please note that, though access restrictions are acceptable now, your entire data will need to be made freely accessible if your manuscript is accepted for publication. This policy applies to all data except where public deposition would breach compliance with the protocol approved by your research ethics board. If you are unable to adhere to our open data policy, please kindly revise your statement to explain your reasoning and we will seek the editor's input on an exemption. Please be assured that, once you have provided your new statement, the assessment of your exemption will not hold up the peer review process.

6. Please amend either the abstract on the online submission form (via Edit Submission) or the abstract in the manuscript so that they are identical.

7. Please include your full ethics statement in the ‘Methods’ section of your manuscript file. In your statement, please include the full name of the IRB or ethics committee who approved or waived your study, as well as whether or not you obtained informed written or verbal consent. If consent was waived for your study, please include this information in your statement as well.

8. Please include a separate caption and explanation for each figure in your manuscript.

9. Please include captions for your Supporting Information files at the end of your manuscript, and update any in-text citations to match accordingly. Please see our Supporting Information guidelines for more information: http://journals.plos.org/plosone/s/supporting-information.

10. If the reviewer comments include a recommendation to cite specific previously published works, please review and evaluate these publications to determine whether they are relevant and should be cited. There is no requirement to cite these works unless the editor has indicated otherwise.

Additional Editor Comments:

Dear Authors the reviewers have raised concerns regarding the the statistical strategy, the definition of outcomes and description study design and how you will address the potential confounders. Kindly address and clarify these issues.

[Note: HTML markup is below. Please do not edit.]

Reviewer's Responses to Questions

**Comments to the Author**

1. Does the manuscript provide a valid rationale for the proposed study, with clearly identified and justified research questions?

Reviewer #1: Yes

Reviewer #2: Partly

Reviewer #3: Yes

**********

2. Is the protocol technically sound and planned in a manner that will lead to a meaningful outcome and allow testing the stated hypotheses?

Reviewer #1: Yes

Reviewer #2: Partly

Reviewer #3: Partly

**********

3. Is the methodology feasible and described in sufficient detail to allow the work to be replicable?

Reviewer #1: Yes

Reviewer #2: Yes

Reviewer #3: No

**********

4. Have the authors described where all data underlying the findings will be made available when the study is complete?

The PLOS Data policy

Reviewer #1: Yes

Reviewer #2: Yes

Reviewer #3: Yes

**********

5. Is the manuscript presented in an intelligible fashion and written in standard English?

Reviewer #1: Yes

Reviewer #2: Yes

Reviewer #3: Yes

**********

Please use the space provided to explain your answers to the questions above and, if applicable, provide comments about issues authors must address before this protocol can be accepted for publication. You may also include additional comments for the author, including concerns about research or publication ethics.

You may also provide optional suggestions and comments to authors that they might find helpful in planning their study.

Reviewer #1: Very well-written protocol using SPIRIT checklist. I am interested in learning the results from your study, this can improve clinical practice with common use of bedside ultrasound. There are two typos in the image section - Inputt, Enrolment. Otherwise, everything looks good.

Reviewer #2: Reviewer Comments to the Authors

Manuscript Title:

Dynamic Assessment of Renal Resistive Index Using Point-of-Care Ultrasonography for Early Detection of Sepsis-Induced Acute Kidney Injury: A Multicenter Clinical Trial Protocol

Overall assessment

This study protocol addresses an important clinical question regarding the use of the renal resistive index (RRI) for early detection of sepsis-associated acute kidney injury. The multicenter design and repeated RRI measurements may provide valuable insights into the dynamic changes of renal hemodynamics in critically ill patients.

However, several methodological aspects require clarification to improve the transparency and robustness of the study design.

Major Comments

1. Clarification of the study design

The manuscript is described as a “multicenter clinical trial protocol.” Based on the methods presented, the study appears to be a prospective observational cohort rather than an interventional clinical trial. The authors should clarify the study design and ensure that the terminology used throughout the manuscript accurately reflects the methodological framework.

2. Rationale for the 7-day monitoring period

The protocol indicates that patients will be followed for seven days after enrollment. Acute kidney injury in patients with sepsis may develop later during hospitalization, and limiting follow-up to seven days could potentially lead to under-detection of AKI events. The authors should justify this follow-up duration or clarify whether AKI occurring later during hospitalization will be captured.

Minor Comments

1. Potential confounding factors affecting RRI

The renal resistive index can be influenced by several systemic and hemodynamic factors in critically ill patients. In addition to mean arterial pressure and heart rate, intra-abdominal pressure, as well as variables such as vasopressor use, mechanical ventilation parameters (e.g., PEEP), and fluid status may also significantly affect RRI values. Clarification is needed regarding whether these factors will be recorded and adjusted for in the statistical analysis.

2. Definition of baseline serum creatinine

The manuscript states that AKI will be diagnosed according to KDIGO 2012 criteria using serum creatinine and urine output. However, the method used to determine baseline serum creatinine is not clearly described, particularly in patients without recent laboratory data prior to admission. The authors should clarify how baseline creatinine will be defined.

3. Clinical implications of RRI monitoring

While the study focuses on the predictive value of RRI for SA-AKI, the manuscript could further clarify how early identification of elevated RRI might influence clinical decision-making in septic patients.

Overall, this protocol addresses an important topic. Clarification of the issues above would improve the methodological transparency of the study.

Reviewer #3: This manuscript outlines the protocol for a multicentre study that evaluates the predictive value of the renal resistive index (RRI), as measured by point-of-care ultrasonography, for the early detection of sepsis-associated acute kidney injury (SA-AKI). This is a clinically relevant topic, as the early detection of AKI in septic patients remains a major challenge in critical care medicine. Using a non-invasive bedside parameter such as the RRI could potentially improve the early stratification of risk and guide clinical decision-making.

Strengths of the proposed study include the multicentre design, dynamic RRI monitoring and simultaneous measurement of several kidney injury biomarkers. However, several important methodological and conceptual issues need to be clarified before the protocol can be adequately evaluated. In particular, further clarification is required on the description of the study design, the statistical strategy, the definition of outcomes and the control of potential confounders influencing RRI.

Below, I provide major and minor comments intended to improve the clarity and methodological rigour of the manuscript.

**********

what does this mean?). If published, this will include your full peer review and any attached files.

If you choose “no”, your identity will remain anonymous but your review may still be made public.

**Do you want your identity to be public for this peer review?**  For information about this choice, including consent withdrawal, please see our Privacy Policy

Reviewer #1: **Yes:**  Karthik Kailasam MD

Reviewer #2: No

Reviewer #3: No

**********

[NOTE: If reviewer comments were submitted as an attachment file, they will be attached to this email and accessible via the submission site. Please log into your account, locate the manuscript record, and check for the action link "View Attachments". If this link does not appear, there are no attachment files.]

To ensure your figures meet our technical requirements, please review our figure guidelines: https://journals.plos.org/plosone/s/figures

You may also use PLOS’s free figure tool, NAAS, to help you prepare publication quality figures: https://journals.plos.org/plosone/s/figures#loc-tools-for-figure-preparation.

NAAS will assess whether your figures meet our technical requirements by comparing each figure against our figure specifications.

Attachment Submitted filename: PONE-D-25-46681_reviewer.pdf

Attachment Submitted filename: Reviewing PLOS ONE.docx

# Author comment: Author response to Decision Letter 1

**DOI:** 10.1371/journal.pone.0359982.r002

25 May 2026

Dear Editor, we have carefully addressed all the comments and made the corresponding revisions in the manuscript.

Attachment Submitted filename: Response to Reviewers.docx

# Peer review: Decision Letter 1

**DOI:** 10.1371/journal.pone.0359982.r003

**Authors:** Ramya Iyadurai

19 Jul 2026

Dear Dr. Ren,

Thank you for submitting your manuscript to PLOS ONE. After careful consideration, we feel that it has merit but does not fully meet PLOS ONE’s publication criteria as it currently stands. Therefore, we invite you to submit a revised version of the manuscript that addresses the points raised during the review process.

==============================

**Dear Authors** **Thank you for your resubmission and making all the changes recommended for your manuscript by the reviewers. Kindly address the reviewer's concerns regarding the biomarkers.**

==============================

Please submit your revised manuscript by Sep 02 2026 11:59PM. If you will need more time than this to complete your revisions, please reply to this message or contact the journal office at plosone@plos.org. When you're ready to submit your revision, log on to https://www.editorialmanager.com/pone/ and select the 'Submissions Needing Revision' folder to locate your manuscript file.

- A letter that responds to each point raised by the academic editor and reviewer(s). You should upload this letter as a separate file labeled 'Response to Reviewers'.
- A marked-up copy of your manuscript that highlights changes made to the original version. You should upload this as a separate file labeled 'Revised Manuscript with Track Changes'.
- An unmarked version of your revised paper without tracked changes. You should upload this as a separate file labeled 'Manuscript'.

If you would like to make changes to your financial disclosure, please include your updated statement in your cover letter. Guidelines for resubmitting your figure files are available below the reviewer comments at the end of this letter.

If applicable, we recommend that you deposit your laboratory protocols in protocols.io to enhance the reproducibility of your results. Protocols.io assigns your protocol its own identifier (DOI) so that it can be cited independently in the future. For instructions see: https://journals.plos.org/plosone/s/submission-guidelines#loc-laboratory-protocols. Additionally, PLOS ONE offers an option for publishing peer-reviewed Lab Protocol articles, which describe protocols hosted on protocols.io. Read more information on sharing protocols at https://plos.org/protocols?utm_medium=editorial-email&utm_source=authorletters&utm_campaign=protocols.

As the corresponding author, your ORCID iD is verified in the submission system and will appear in the published article. PLOS supports the use of ORCID, and we encourage all coauthors to register for an ORCID iD and use it as well. Please encourage your coauthors to verify their ORCID iD within the submission system before final acceptance, as unverified ORCID iDs will not appear in the published article. *Only*  the individual author can complete the verification step; PLOS staff *cannot*  verify ORCID iDs on behalf of authors.

We look forward to receiving your revised manuscript.

Kind regards,

Ramya Iyadurai

Academic Editor

PLOS One

Journal Requirements:

If the reviewer comments include a recommendation to cite specific previously published works, please review and evaluate these publications to determine whether they are relevant and should be cited. There is no requirement to cite these works unless the editor has indicated otherwise.

Please review your reference list to ensure that it is complete and correct. If you have cited papers that have been retracted, please include the rationale for doing so in the manuscript text, or remove these references and replace them with relevant current references. Any changes to the reference list should be mentioned in the rebuttal letter that accompanies your revised manuscript. If you need to cite a retracted article, indicate the article’s retracted status in the References list and also include a citation and full reference for the retraction notice.

[Note: HTML markup is below. Please do not edit.]

Reviewers' comments:

Reviewer's Responses to Questions

**Comments to the Author**

1. Does the manuscript provide a valid rationale for the proposed study, with clearly identified and justified research questions?

Reviewer #4: Yes

**********

2. Is the protocol technically sound and planned in a manner that will lead to a meaningful outcome and allow testing the stated hypotheses?

Reviewer #4: Yes

**********

3. Is the methodology feasible and described in sufficient detail to allow the work to be replicable?

Reviewer #4: Yes

**********

4. Have the authors described where all data underlying the findings will be made available when the study is complete?

The PLOS Data policy

Reviewer #4: Yes

**********

5. Is the manuscript presented in an intelligible fashion and written in standard English?

Reviewer #4: Yes

**********

Please use the space provided to explain your answers to the questions above and, if applicable, provide comments about issues authors must address before this protocol can be accepted for publication. You may also include additional comments for the author, including concerns about research or publication ethics.

You may also provide optional suggestions and comments to authors that they might find helpful in planning their study.

Reviewer #4: Overall Evaluation

This manuscript is a study protocol with ongoing patient recruitment, so complete quantitative clinical outcome data are not available, which conforms to PLOS ONE’s publication standards for protocol manuscripts. This study focuses on dynamic bedside ultrasound monitoring of renal resistive index for early identification of sepsis-associated acute kidney injury, with remarkable clinical value. The study design, sample size calculation, standardized monitoring schedule, confounding factor adjustment and statistical analysis plan are rigorous and well-organized. The authors have fully responded to all comments from the previous three reviewers and implemented corresponding revisions. The manuscript is well-structured and logically consistent, and generally satisfies the journal’s publication requirements. Only two minor flaws in figures need to be addressed.

Minor Revisions Required

1.Inconsistent biomarker information between the main text and Figure 3 The main text clearly states that NGAL will not be tested in this study, whereas the timeline table in Figure 3 still contains items related to NGAL. This inconsistency may confuse readers. The authors should remove all NGAL-related entries from Figure 3.

2.Uncorrected spelling error in Figure 3 The authors have revised the misspellings “Inputt” and “Enrolment”, and the typo “Informed concent” in Figure 1 has also been fixed. Nevertheless, the same spelling error remains in Figure 3: the phrase “Informed concent” is incorrect, and the standard clinical term is “Informed consent”.

3.Incomplete Data Availability Statement The text only mentions that all relevant research data will be released after trial completion, without specifying the repository, storage location or access channel for the final dataset. In accordance with PLOS ONE’s open data policy, the authors should add detailed information about where the complete trial data will be archived and accessed once recruitment finishes.

Final Recommendation

Apart from the two trivial figure-related issues mentioned above, this protocol features complete scientific logic, standardized implementation procedures and sound statistical design. All major methodological and scientific concerns raised in the prior review round have been properly resolved. I recommend acceptance after the author completes the two revisions for figures.

**********

what does this mean?). If published, this will include your full peer review and any attached files.

If you choose “no”, your identity will remain anonymous but your review may still be made public.

**Do you want your identity to be public for this peer review?**  For information about this choice, including consent withdrawal, please see our Privacy Policy

Reviewer #4: No

**********

[NOTE: If reviewer comments were submitted as an attachment file, they will be attached to this email and accessible via the submission site. Please log into your account, locate the manuscript record, and check for the action link "View Attachments". If this link does not appear, there are no attachment files.]

To ensure your figures meet our technical requirements, please review our figure guidelines: https://journals.plos.org/plosone/s/figures

You may also use PLOS’s free figure tool, NAAS, to help you prepare publication quality figures: https://journals.plos.org/plosone/s/figures#loc-tools-for-figure-preparation.

NAAS will assess whether your figures meet our technical requirements by comparing each figure against our figure specifications.

# Author comment: Author response to Decision Letter 2

**DOI:** 10.1371/journal.pone.0359982.r004

15 Sep 2026

Minor Revisions Required

1. Inconsistent biomarker information between the main text and Figure 3 The main text clearly states that NGAL will not be tested in this study, whereas the timeline table in Figure 3 still contains items related to NGAL. This inconsistency may confuse readers. The authors should remove all NGAL-related entries from Figure 3.

Answer:Thank you for your valuable comment. We have removed all NGAL-related entries from Figure 3.

2. Uncorrected spelling error in Figure 3 The authors have revised the misspellings “Inputt” and “Enrolment”, and the typo “Informed concent” in Figure 1 has also been fixed. Nevertheless, the same spelling error remains in Figure 3: the phrase “Informed concent” is incorrect, and the standard clinical term is “Informed consent”.

Answer:Thank you for your valuable comment. We have corrected the spelling error in Figure 3. The corresponding revision has been made in the revised manuscript.

3. Incomplete Data Availability Statement The text only mentions that all relevant research data will be released after trial completion, without specifying the repository, storage location or access channel for the final dataset. In accordance with PLOS ONE’s open data policy, the authors should add detailed information about where the complete trial data will be archived and accessed once recruitment finishes.

Answer: Thank you for your valuable comment. We appreciate your suggestion regarding the Data Availability Statement. The relevant research data will be deposited in the Figshare public repository upon completion of this clinical trial and publication of the study. The dataset will be freely available through a permanent DOI, which will be included in the final published article.

Attachment Submitted filename: Response_to_Reviewers_auresp_2.docx

# Editor report: Decision Letter 2

**DOI:** 10.1371/journal.pone.0359982.r005

**Authors:** Ramya Iyadurai

21 Sep 2026

Dynamic Assessment of Renal Resistive Index Using Point-of-Care Ultrasonography for Early Detection of Sepsis-Induced Acute Kidney Injury: A Multicenter Clinical Trial Protocol

PONE-D-25-46681R2

Dear Dr. Ren,

We’re pleased to inform you that your manuscript has been judged scientifically suitable for publication and will be formally accepted for publication once it meets all outstanding technical requirements.

Within one week, you’ll receive an e-mail detailing the required amendments. When these have been addressed, you’ll receive a formal acceptance letter and your manuscript will be scheduled for publication.

An invoice will be generated when your article is formally accepted. Please note, if your institution has a publishing partnership with PLOS and your article meets the relevant criteria, all or part of your publication costs will be covered. Please make sure your user information is up-to-date by logging into Editorial Manager at Editorial Manager® and clicking the ‘Update My Information' link at the top of the page. For questions related to billing, please contact billing support.

If your institution or institutions have a press office, please notify them about your upcoming paper to help maximize its impact. If they’ll be preparing press materials, please inform our press team as soon as possible -- no later than 48 hours after receiving the formal acceptance. Your manuscript will remain under strict press embargo until 2 pm Eastern Time on the date of publication. For more information, please contact onepress@plos.org.

Kind regards,

Ramya Iyadurai

Academic Editor

PLOS One

Additional Editor Comments (optional):

Reviewers' comments:

# Editor report: Acceptance letter

**DOI:** 10.1371/journal.pone.0359982.r006

**Authors:** Ramya Iyadurai

PONE-D-25-46681R2

PLOS One

Dear Dr. Ren,

I'm pleased to inform you that your manuscript has been deemed suitable for publication in PLOS One. Congratulations! Your manuscript is now being handed over to our production team.

At this stage, our production department will prepare your paper for publication. This includes ensuring the following:

* All references, tables, and figures are properly cited

* All relevant supporting information is included in the manuscript submission,

* There are no issues that prevent the paper from being properly typeset

You will receive further instructions from the production team, including instructions on how to review your proof when it is ready. Please keep in mind that we are working through a large volume of accepted articles, so please give us a few days to review your paper and let you know the next and final steps.

Lastly, if your institution or institutions have a press office, please let them know about your upcoming paper now to help maximize its impact. If they'll be preparing press materials, please inform our press team within the next 48 hours. Your manuscript will remain under strict press embargo until 2 pm Eastern Time on the date of publication. For more information, please contact onepress@plos.org.

You will receive an invoice from PLOS for your publication fee after your manuscript has reached the completed accept phase. If you receive an email requesting payment before acceptance or for any other service, this may be a phishing scheme. Learn how to identify phishing emails and protect your accounts at https://explore.plos.org/phishing.

If we can help with anything else, please email us at customercare@plos.org.

Thank you for submitting your work to PLOS One and supporting open access.

Kind regards,

PLOS One Editorial Office Staff

on behalf of

Dr. Ramya Iyadurai

Academic Editor

PLOS One
